# Transgenerational effect of mutants in the RNA-directed DNA methylation pathway on the triploid block in *Arabidopsis*

**DOI:** 10.1186/s13059-021-02359-2

**Published:** 2021-05-06

**Authors:** Zhenxing Wang, Nicolas Butel, Juan Santos-González, Lauriane Simon, Cecilia Wärdig, Claudia Köhler

**Affiliations:** 1 Department of Plant Biology, Swedish University of Agricultural Sciences and Linnean Center for Plant Biology, 75007 Uppsala, Sweden; 2 Present address: College of Horticulture, Nanjing Agricultural University and State Key Laboratory of Crop Genetics and Germplasm Enhancement, Nanjing, 210095 China; 3 Max Planck Institute of Molecular Plant Physiology, Am Mühlenberg 1, 14476 Potsdam-Golm, Germany

## Abstract

**Background:**

Hybridization of plants that differ in number of chromosome sets (ploidy) frequently causes endosperm failure and seed arrest, a phenomenon referred to as triploid block. In *Arabidopsis*, loss of function of *NRPD1*, encoding the largest subunit of the plant-specific RNA polymerase IV (Pol IV), can suppress the triploid block. Pol IV generates short RNAs required to guide de novo methylation in the RNA-directed DNA methylation (RdDM) pathway. Recent work suggests that suppression of the triploid block by mutants in RdDM components differs, depending on whether the diploid pollen is derived from tetraploid plants or from the *omission in second division 1* (*osd1*) mutant. This study aims to understand this difference.

**Results:**

In this study, we find that the ability of mutants in the RdDM pathway to suppress the triploid block depends on their degree of inbreeding. While first homozygous generation mutants in RdDM components *NRPD1*, *RDR2*, *NRPE1*, and *DRM2* have weak or no ability to rescue the triploid block, they are able to suppress the triploid block with successive generations of inbreeding. Inbreeding of *nrpd1* was connected with a transgenerational loss of non-CG DNA methylation on sites jointly regulated by CHROMOMETHYLASES 2 and 3.

**Conclusions:**

Our data reveal that loss of RdDM function differs in its effect in early and late generations, which has important implications when interpreting the effect of RdDM mutants.

**Supplementary Information:**

The online version contains supplementary material available at 10.1186/s13059-021-02359-2.

## Introduction

Hybridization of plants that differ in ploidy frequently leads to seed arrest, a phenomenon referred to as the triploid block [1, 2]. The triploid block is established in the endosperm, a nutritive tissue supporting embryo growth [3–5]. The endosperm is typically a triploid tissue, derived after fertilization of the diploid central cell by one of the sperm cells [6]. In most flowering plant species, the endosperm initially develops as a coenocyte and undergoes cellularization after a defined number of nuclear divisions [7]. In *Arabidopsis thaliana*, as in many other flowering plant species, hybridizations of maternal plants with higher ploidy pollen donors cause failure of the endosperm to cellularize, leading to embryo arrest [8, 9]. Sensitivity of the endosperm to parental genome dosage is closely connected to genomic imprinting, an epigenetic phenomenon resulting in the parental-specific expression of specific genes [10, 11]. Specifically, loss of function of the imprinted paternally expressed genes (PEGs) *ADMETOS*, *SUVH7*, *SUVH9*, *AHL10*, *PEG2*, *PEG9*, *PICKLE RELATED2* (*PKR2*), and *PHERES1* (*PHE1*) is sufficient to suppress the triploid block [12–14], suggesting a causal role of imprinted genes in establishing the triploid block. Similarly, loss of function of the paternally biased *NRPD1* gene, encoding the largest subunit of the plant-specific RNA polymerase IV (Pol IV), leads to suppression of the triploid block [15, 16]. Pol IV is a central component of the RNA-directed DNA methylation (RdDM) pathway that establishes DNA methylation in all sequence contexts and maintains CHH methylation (H corresponds to A, C, or T) preferentially on small euchromatic TEs [17, 18]. Pol IV forms short transcripts of 26–45 nt in size that are converted into double-stranded RNA by the RNA-DEPENDENT RNA POLYMERASE 2 [19, 20]. Double-stranded RNAs are then targeted by different DICER-LIKE (DCL) proteins to generate small RNAs (sRNAs) in the size range of 21–24 nt that are incorporated into ARGONAUTE (AGO) proteins. These sRNA-AGO complexes pair with Pol V-derived scaffold transcripts and recruit the DOMAINS REARRANGED METHYLTRANSFERASE2 (DRM2), which methylates DNA in all sequence contexts [18, 21–23]. Pol IV is recruited to heterochromatic regions by SAWADEE HOMEODOMAIN HOMOLOGUE 1 (SHH1), which recognizes dimethylated histone H3 lysine 9 (H3K9me2) [24, 25]. Methylation on CHH positions can also be mediated by CHROMOMETHYLASE 2 (CMT2), which acts in a feedback loop with H3K9me2 [26, 27]. CMT2 can also target CHG positions, but at reduced efficiency [27]. The main CHG methyltransferase is CHROMOMETHYLASE 3 (CMT3), which like CMT2 maintains CHG methylation in a feedback loop with H3K9me2 [26, 28–31]. Both CMT2 and CMT3 preferentially target heterochromatic TEs, while the RdDM pathway preferentially targets short euchromatic TEs [26]. Maintenance of CG methylation requires METHYLTRANSFERASE 1 (MET1), which recognizes hemi-methylated symmetrical CG nucleotides [18, 21]. Loss of paternal MET1 function suppresses the triploid block [32], similar to aforementioned mutants in PEGs. Also, the triple *suvh4/5/6* mutant that is deficient in the H3K9me2 methyltransferases KRYPTONITE (KYP, or SUVH4) and the redundantly acting SUVH5 and SUVH6 is a strong suppressor of the triploid block [33]. These data point that there is a connection between DNA methylation and the triploid block, but the precise mechanisms and targets remain to be identified.

Recent work suggests that suppression of the triploid block by mutants in the RdDM components *RDR2*, *DCL3*, *NRPE1*, and *DRM2* differs, depending on whether the diploid pollen (2n) is derived from tetraploid (4x) plants or from the *omission in second division 1* (*osd1*) mutant. Loss of *OSD1* causes an omission of the second meiotic division, leading to 2n pollen formation [34]. While 4x mutants in *NRPE1*, *RDR2*, *DCL3*, and *DRM2* suppress the triploid block [35], no suppressive effect of those mutants was found in the *osd1* background [16]. However, mutants in the Pol IV component *NRPD1* could similarly suppress the triploid block in 4x and *osd1* backgrounds [16, 35], suggesting that there is a difference in the response to loss of RdDM function in *osd1* and 4x plants. Tetraploid RdDM mutants were generated from inbred mutants using colchicine treatment [35], while RdDM mutants in *osd1* background were selected from segregating F2 populations [16]. Previous work in maize revealed that loss of Pol IV function causes a progressively enhanced loss of silencing over generations [36], suggesting that it could make a difference whether using first-generation homozygous RdDM mutants or highly inbred mutants. In this study, we challenged this hypothesis by testing whether inbreeding does enhance the suppressive effect of RdDM mutants in the *osd1* background. We report that inbred mutants in *nrpd1*, *nrpe1*, and *drm2* have a successively enhanced ability to suppress the triploid block. Inbreeding of *nrpd1* was connected to a transgenerational loss of non-CG DNA methylation on sites jointly regulated by CHROMOMETHYLASES 2 and 3 (CMT2/3). Our data thus reveal that loss of RdDM function differs in its effect in early and late generations, highlighting the importance of tracking generations when interpreting effects of RdDM mutants.

## Results

### Inbreeding of RdDM mutants enhanced their ability to rescue the triploid block

**Fig. 1:**
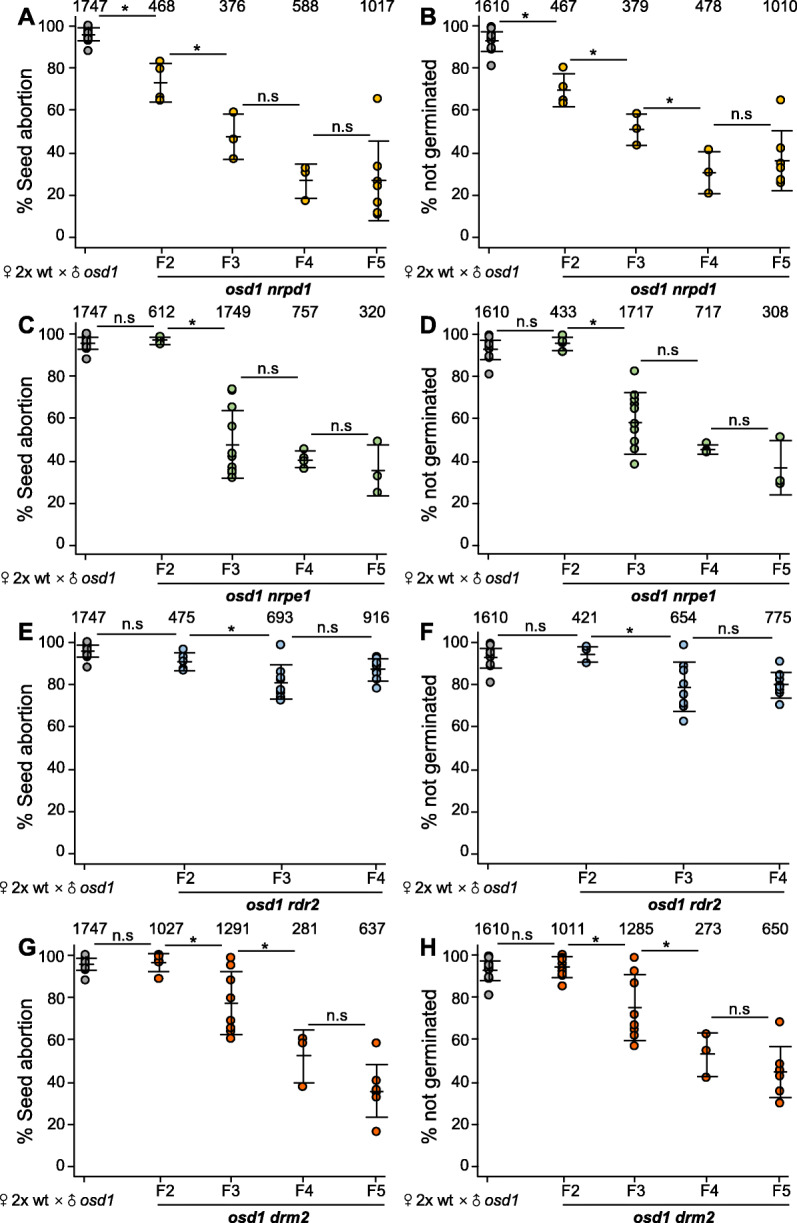
Inbreeding of RdDM *osd1* mutants enhanced their ability to rescue the triploid block. **a**, **c**, **e**, **g** Seed abortion rates of homozygous *osd1* or RdDM *osd1* double homozygous mutants crossed as pollen donors to diploid wild-type plants. **b**, **d**, **f**, **h** The percentage of seeds that failed to germinate from each cross. Each filled circle represents 2–4 siliques from a single inflorescence pooled as one cross. Numbers on top represent total seed numbers. Asterisk represents statistically significant difference (*p* < 0.05) in comparison between the indicated groups. n.s, not significant. Statistical significance calculated by ANOVA with post hoc Tukey’s HSD test

The reported difference in the ability to suppress the triploid block between 4x RdDM mutants [35] and RdDM *osd1* double mutants [16] could be a consequence of different plant growth conditions or due to the different genetic backgrounds. To distinguish between both possibilities, we tested the 4x RdDM mutants for their ability to suppress the triploid block when grown under our conditions. The suppressive effect of 4x *nrpe1* and 4x *drm2* was as strong as the reported effect of *osd1 nrpd1* and 4x *nrpd1* [16, 35], while the suppressive effect for 4x *dcl3* and 4x *rdr2* was weaker (Additional file 1: Figure S1), consistent with previous work [35]. Thus, different growth conditions could not explain the difference between the results obtained with RdDM mutants in the *osd1* or 4x background. One possible explanation for this difference could be inbreeding; while 4x RdDM mutants were generated by colchicine treatment of inbred RdDM mutants, RdDM mutants in the *osd1* background were tested after the first generation of homozygosity. To test this hypothesis, we analyzed whether inbreeding of RdDM mutants in the *osd1* background would change their ability to suppress the triploid block (Additional file 1: Figure S2). We found that inbreeding indeed significantly increased the suppressive effect of *osd1 nrpd1*, *osd1 nrpe1*, and *osd1 drm2*, but had only a weak effect on *osd1 rdr2* (Fig. 1). Interestingly, only *osd1 nrpd1* had a significant suppressive effect in the F2 generation (first generation of *nrpd1* homozygosity), while other RdDM pathway mutants only had an effect in the F3 and later generations.

### Inbreeding of *nrpd1* caused increased loss of DNA methylation

**Fig. 2:**
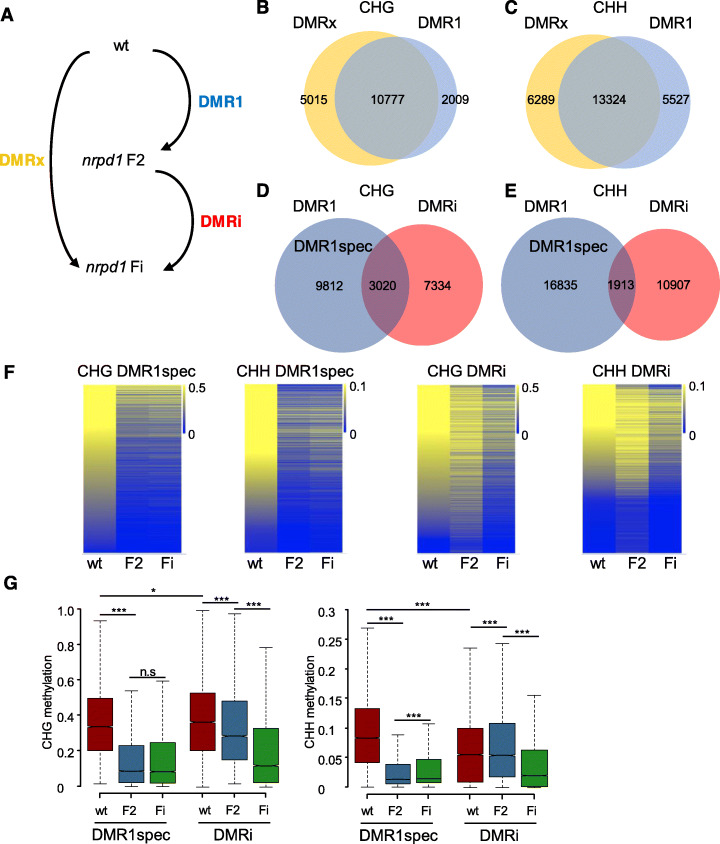
Transgenerational change of non-CG DNA methylation in inbred generations of *nrpd1* mutants. **a** Scheme of defining three groups of differentially methylated regions (DMRs): wild type (wt), first-generation homozygous *nrpd1* (F2), and inbred *nrpd1* (Fi). **b**, **c** Venn diagrams showing the overlap of CHG DMRx and DMR1 (**b**) and CHH DMRx and DMR1 (**c**). **d**, **e** Venn diagrams showing the overlap of CHG DMR1 and DMRi (**d**) and CHH DMR1 and DMRi (**e**). DMR1spec refers to DMR1 not overlapping with DMRi. **f** Heatmaps of fractional CHG and CHH DNA methylation levels at DMR1spec and DMRi loci in wt, F2, and Fi *nrpd1* mutants. **g** Boxplots of fractional CHG and CHH DNA methylation levels at DMR1spec and DMRi loci in wt, F2, and Fi *nrpd1* mutants. Boxes show medians and the interquartile range, and error bars show the full range excluding outliers. *p < 0.05, ***p < 0.001; n.s, not significant (Wilcoxon test)

Since the suppressive effect of the triploid block by *osd1 nrpd1*, *osd1 nrpe1*, and *osd1 drm2* became stronger with increasing number of generations, we suspected that there is a transgenerational change of DNA methylation from F2 to higher inbred generations of *nrpd1*. We tested this hypothesis by generating bisulfite sequencing data of first-generation homozygous *nrpd1* mutants segregating in an F2 population and higher inbred generations of *nrpd1* (three times inbred, denoted as Fi; Additional file 1: Figure S3) and tested for differences in DNA methylation (Fig. 2a; Additional file 2: Table S1). Since CHH methylation levels are developmentally regulated in the endosperm [37] and thus prone to potential variability, we analyzed leaf tissue of F2 and inbred (Fi) *nrpd1* mutants. The RdDM pathway targets cytosines in all sequence contexts, but has its main effects on non-CG methylation [27]. We therefore focused on transgenerational changes in CHG and CHH methylation. We identified differentially methylated regions (DMRs) that were hypomethylated in first-generation homozygous *nrpd1* (F2) mutants compared to wild type (referred to as DMR1, see Fig. 2a, Additional file 3: Table S2, Additional file 8: Table S7), hypomethylated DMRs between F2 and inbred (Fi) *nrpd1* mutants (referred to as DMRi, Additional file 3: Table S2, Additional file 8: Table S7), and hypomethylated DMRs between *nrpd1* Fi mutants and wild type (referred to as DMRx, Additional file 3: Table S2, Additional file 8: Table S7). As expected, most DMR1 were also detectable in inbred generations (DMRx) (Fig. 2a–c). However, we found that inbred generations of *nrpd1* mutants gained many DMRs in CHG and CHH sequence contexts (DMRi, Fig. 2d, e).

Those DMR1 regions that were not affected by inbreeding and were thus not overlapping with DMRi regions were defined as DMR1spec (Fig. 2d, e) and compared to DMRi. Visualization of DMR1spec and DMRi using heatmaps and boxplots revealed instant loss of DNA methylation in the first generation of *nrpd1* homozygous mutants for DMR1spec and gradual loss of DNA methylation upon inbreeding for DMRi (Fig. 2f, g), consistent with the defining criteria for DMR1spec and DMRi.

In wild-type plants, methylation levels in CHG context were slightly higher in DMRi compared to DMR1spec, while CHH methylation levels were significantly lower in DMRi compared to DMR1spec (Fig. 2g), suggesting differential activity of the RdDM pathway on both types of DMRs. Nearly half of DMR1spec and DMRi associated with TEs, and the other half associated with promoter and coding regions (Additional file 1: Figure S4A). There were significant differences in the association of both types of DMRs to genic regions; DMRi were more frequently associated with coding regions than DMR1spec, and conversely, DMR1spec were more frequently associated with promoter regions than DMRi (Additional file 1: Figure S4A). DMR1spec and DMRi were preferentially associated with different TE families; CHG and CHH DMR1spec were more frequently associated with helitrons, but depleted in Gypsy and Copia TEs. Conversely, DMRi were more frequently associated with Gypsy TEs, but depleted on helitrons (Additional file 1: Figure S4B).

### Loss of RdDM differentially affects DMR1spec and DMRi

**Fig. 3:**
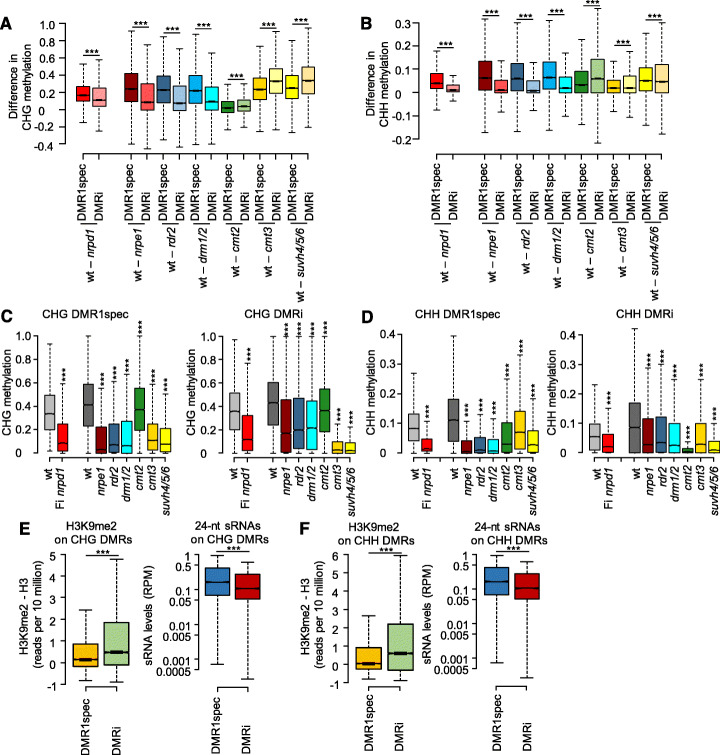
DMR1spec and DMRi are differentially affected by loss of RdDM and other DNA methylation pathways. **a** Boxplots showing loss of fractional CHG methylation in mutants compared to wild type (wt - mutant) on DMR1spec and DMRi. **b** Boxplots showing loss of fractional CHH methylation in mutants compared to wt (wt - mutant) on DMR1spec and DMRi. **c** Boxplots of fractional CHG methylation levels on DMR1spec and DMRi in wild type (wt) and Fi *nrpd1* (data generated in this study) and DNA methylation mutants (data generated in [38]). **d** Boxplots of fractional CHH methylation levels on DMR1spec and DMRi in wt and Fi *nrpd1* (data generated in this study) and DNA methylation mutants (data generated in [38]). **e** Boxplots of H3K9me2 and 24-nt siRNA levels on CHG DMR1spec and DMRi in wt leaves. **f** Boxplots of H3K9me2 and 24-nt siRNA levels on CHH DMR1spec and DMRi in wt leaves. Boxes show medians and the interquartile range, and error bars show the full range excluding outliers. ***p < 0.001 (Wilcoxon test). The statistical tests were performed between indicated mutants and wt in **c** and **d**, and the indicated pairs in **a**, **b**, **e**, and **f**. For definition of DMRs, see Fig. 2a

Using published bisulfite data of various mutants in RdDM components and other DNA methylation pathways [38], we tested whether DMR1spec and DMRi were differentially affected by loss of different silencing pathways. Indeed, DMR1spec and DMRi differed in their response to loss of RdDM pathway mutants; loss of CHG and CHH methylation was significantly stronger in *nrpd1*, *nrpe1*, *rdr2*, and *drm1/2* on DMR1spec than on DMRi (Fig. 3a–d). These data indicate that DMR1spec differ from DMRi in their dependency on RdDM and that methylation at DMRi is redundantly maintained by RdDM and other mechanisms. Previous work revealed that CHG and CHH methylation is partially redundantly regulated by all non-CG methyltransferases, which include DRM2, CMT2, and CMT3 [27]. We therefore analyzed CHG and CHH methylation on DMR1spec and DMRi in *cmt2* and *cmt3* mutant backgrounds (Fig. 3a–d). Since CMT2 and CMT3 are recruited by H3K9me2 [29, 31, 39], we included the H3K9me2 depleted *suvh4/5/6* triple mutant in this analysis. Consistent with the idea that DMRi is redundantly targeted by other DNA methylation pathways, we found that DMRi experienced a significantly stronger loss of CHG methylation in *cmt3* and *suvh4/5/6* mutant backgrounds compared to DMR1spec (Fig. 3a). Similarly, CHH methylation levels in DMRi were significantly stronger affected by loss of *CMT2* than in DMR1spec (Fig. 3b). Nevertheless, despite the stronger effect of *suh4/5/6*, *cmt3*, and *cmt2* on DMRi, also DMR1spec were significantly affected in those mutants (Fig. 3c, d). Preferential targeting of DMRi by CMT2 and CMT3 pathways correlated with significantly higher levels of H3K9me2 on DMRi compared to DMR1spec (Fig. 3e, f). Conversely, DMR1spec had significantly higher levels of 24-nt sRNAs compared to DMRi (Fig. 3e, f), correlating with their preferential targeting by the RdDM pathway. Together, we conclude that DMR1spec and DMRi are redundantly targeted by RdDM, CMT2, and CMT3 pathways (Fig. 3c, d). While loss of RdDM components had a stronger effect on DMR1spec, DMRi were more strongly affected by loss of the CMT2/CMT3 pathway, providing a possible explanation for the persistent DNA methylation on DMRi upon initial loss of the RdDM pathway.

### DMRi overlap with deregulated genes in triploid seeds

**Fig. 4:**
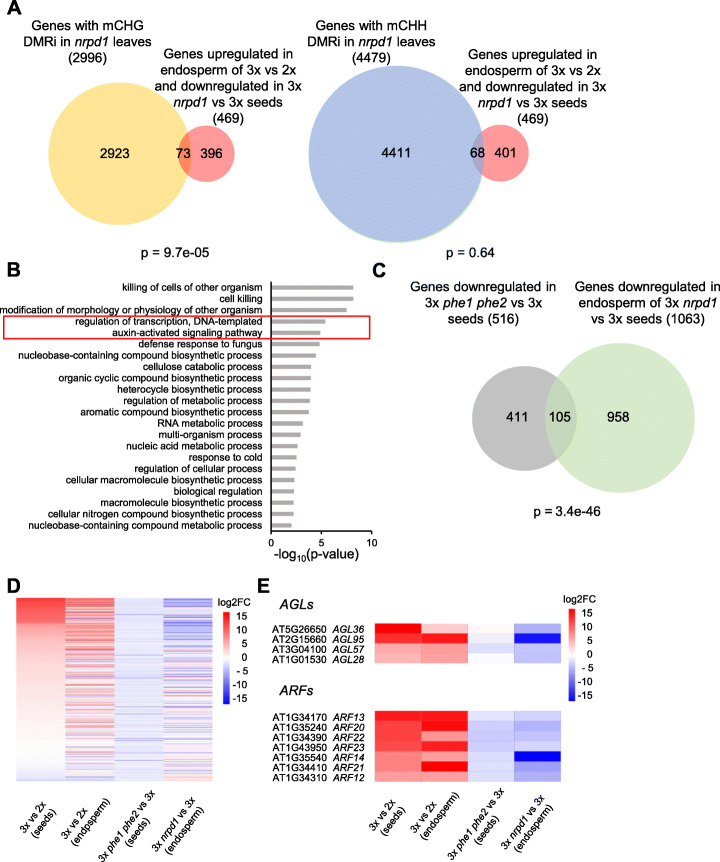
Overlap of DMRi with deregulated genes in triploid (3x) seeds. **a** Venn diagrams showing overlap of non-CG DMRi intersected genes and deregulated genes in *Arabidopsis* endosperm of 3x seeds (log2FC > 1, *p* < 0.05 in 3x vs 2x wt and log2FC < −1, *p* <0.05 in 3x *nrpd1* vs 3x) [16]. **b** Enriched gene ontologies (GO) for biological processes (*p* < 0.01) of intersected CHG DMRi overlapping genes and deregulated genes in *Arabidopsis* endosperm of 3x seeds (log2FC > 1, *p* < 0.05 in 3x vs 2x wt and log2FC < −1, *p* <0.05 in 3x *nrpd1* vs 3x). **c** Venn diagram showing overlap of genes downregulated (log2FC < −1, *p* <0.05) in 3x *phe1 phe2* seeds vs 3x seeds [40] and genes downregulated (log2FC < −1, *p* <0.05) in endosperm of 3x *nrpd1* vs 3x seeds [16]. **d** Heatmap showing genes downregulated (log2FC < −1, *p* <0.05) in 3x *phe1 phe2* seeds vs 3x seeds [40] and their expression in the endosperm of 3x vs 2x seeds [16], 3x *phe1 phe2* vs 3x seeds, and endosperm of 3x *nrpd1* vs 3x seeds [16]. **e** Heatmap of type I *AGLs* and *ARFs* with CHG DMRi and upregulated (log2FC > 1, *p* <0.05) in the endosperm of 3x vs 2x seeds [16] and their expression in 3x vs 2x and 3x *phe1 phe2* vs 3x seeds [40] and endosperm of 3x *nrpd1* vs 3x seeds. For definition of DMRs, see Fig. 2a

The *osd1 nrpd1* mutant was able to suppress the triploid block in the first homozygous generation, but the suppressive effect was strongly enhanced by increasing generations of inbreeding (Fig. 1). Similarly, *osd1 nrpe1* and *osd1 drm2* gained the ability to suppress the triploid block after successive generations of inbreeding. One possible explanation for this phenomenon is that causal loci affected by NRPD1 lose DNA methylation with increasing generations of inbreeding. To test this hypothesis, we identified genes overlapping DMRi and analyzed their expression in triploid seeds. We found a significant overlap of genes that were upregulated in the endosperm of 3x versus 2x seeds (log2FC > 1, *p* < 0.05) and downregulated in the endosperm of 3x *nrpd1* versus 3x seeds (log2FC < −1, *p* < 0.05) (data source [16]) with those having a CHG DMRi in their vicinity (within 1kb of promoter and coding region) (Fig. 4a, Additional file 4: Table S3). Gene Ontology (GO) analysis showed that those genes overlapping with CHG DMRi were strongly enriched for transcription factors, in particular type I AGAMOUS-LIKE (AGL) transcription factors and AUXIN RESPONSE FACTORS (ARFs) (Fig. 4b, Additional file 4: Table S3). While the overlap of deregulated genes with CHH DMRi was not significant (Fig. 4a), the overlapping genes also included AGLs and ARFs (Additional file 4: Table S3). Among the *AGL* genes was *AGL28* (overlapped with CHG DMRi), which encodes a potential interaction partner for PHERES1 (PHE1) and *AGL36* (overlapped with CHH DMRi), a close ortholog of *PHE1* that may possibly act redundantly with *PHE1* [40, 41]. Loss of PHE1 function can suppress the triploid block [40], suggesting that the regulation of AGLs by Pol IV-derived siRNAs may be functionally relevant. In support of this notion, we found a significant overlap of genes downregulated in triploid *phe1 phe2* seeds with downregulated genes in 3x *nrpd1* seeds (Fig. 4c–e, Additional file 1: Figure S5).

### Genes marked by DMRi undergo DNA methylation changes in the endosperm of 3x seeds

**Fig. 5:**
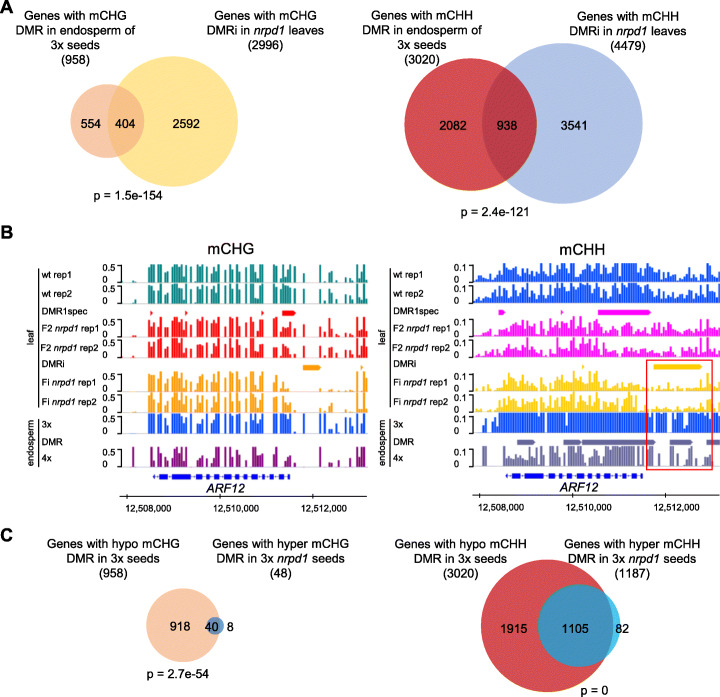
DMRi associated with genes losing DNA methylation in the endosperm of 3x seeds. **a** Venn diagrams showing overlap of genes with hypo non-CG DMRs in the endosperm of 3x seeds [35] and genes with non-CG DMRi in *nrpd1* leaves. **b** *ARF12* losing non-CG methylation during generations in *nrpd1* leaves and endosperm of 3x seeds [35]. The representative region is highlighted by a red box. DMRs are marked by bars. **c** Venn diagrams showing overlap of genes with hypo non-CG DMRs in the endosperm of 3x vs 2x seeds and hyper DMRs in the endosperm of 3x *nrpd1* vs 3x seeds [35]. For definition of DMRs, see Fig. 2a

We tested whether genes overlapping with DMRi in leaves would be similarly marked by DMRs in the endosperm of 3x versus 2x seeds. The endosperm of 3x seeds has reduced CHH methylation [16, 32, 35]. Using previously published data [35], we identified genes overlapping with hypomethylated DMRs (hypo DMRs) in the endosperm of 3x versus 2x. A significant number of genes that overlapped with CHG and CHH DMRi in *nrpd1* leaves also had CHG and CHH hypo DMRs in the endosperm of 3x seeds (Fig. 5a). Importantly, among those genes were *AGL28*, *AGL36*, and *ARFs 12-14*, *20-23* (Fig. 5b, Additional file 1: Figure S6, Additional file 5: Table S4). Previous work revealed that CHH methylation loss in the endosperm of triploid seeds is partly restored upon pollination with *nrpd1* pollen [16, 35]. Using previously published data [35], we identified genes overlapping with hypermethylated DMRs (hyper DMRs) in 3x *nrpd1* versus 3x seeds (Additional file 6: Table S5). A significant number of genes marked by CHH hypoDMRs in the endosperm of 3x seeds gained CHH methylation in the endosperm of 3x *nrpd1* seeds (Fig. 5c), among those *ARFs 12*, *14*, *15*, *20-23* (Additional file 6: Table S5). Thus, genes marked by DMRi in leaves lose DNA methylation in the endosperm of triploid seeds, correlating with increased expression. Conversely, loss of paternal *NRPD1* causes increased DNA methylation in the endosperm, correlating with repression of many genes. The mechanism leading to increased DNA methylation upon loss of paternal *NRPD1* and whether the effect on gene expression is a direct or rather an indirect consequence remains to be established.

Together, our data reveal that inbreeding of *nrpd1*, *nrpe1*, and *drm2* in the *osd1* background triggered increased suppression of the triploid block. Inbreeding of *nrpd1* caused increasing loss of CHG and CHH methylation at defined loci, providing a possible explanation for the enhanced suppressive effect of RdDM mutants over generations.

## Discussion

In this study, we report that mutations in the RdDM components *NRPD1*, *NRPE1*, and *DRM2* triggered a progressively enhanced suppression of the triploid block over generations of inbreeding. Inbreeding of *nrpd1* caused an increasing loss of CHH and CHG methylation over generations, suggesting that the generation-dependent suppression of the triploid block connects to a generation-dependent loss of DNA methylation. Previous work revealed that some loci demethylated in a *nrpd1* background do not regain their original methylation level after restoration of NRPD1 function [42]. The gradual suppression of the triploid block through RdDM mutants, however, strongly suggests that the loci involved in the triploid block can be efficiently reset upon restored RdDM function.

Loci showing increasing loss of CHG and CHH methylation over generations (DMRi) were marked by higher levels of H3K9me2 and were more strongly affected in *cmt2* and *cmt3* mutants than loci that lost DNA methylation in the first homozygous generation (DMR1spec, Fig. 1a, d) of *nrpd1*. This suggests that DMRi loci are partly redundantly targeted by RdDM, CMT2, and CMT3 pathways and that upon loss of RdDM the efficiency of CMT2 and CMT3 to maintain methylation on those loci decreases over generations. The *suvh4/5/6* triple mutant has a strong suppressive effect on the triploid block [33], supporting a possible redundancy of CMT3 and CMT2 pathways on functionally relevant loci. Cooperation of all non-CG methyltransferases to regulate CHG and CHH methylation was previously demonstrated in *Arabidopsis* [27], adding support to this notion. The difference between DMRs that rapidly lose DNA methylation in *nrpd1* (DMR1spec) versus those that maintain DNA methylation over several generations in the absence of RdDM (DMRi) might be linked to a different contribution of the RdDM pathway and CMT2/CMT3 to DNA methylation at particular loci. In line with previous findings [43], we propose that DMR1spec are loci recently targeted by RdDM and that therefore strongly rely on RdDM to maintain DNA methylation, whereas DMRi are possibly ancient RdDM targeted loci transiting from RdDM to an RdDM-independent maintenance phase involving CMT2/CMT3. This transition state could explain why the CMT2/CMT3 pathway is not sufficient to maintain DNA methylation at those loci.

We consider two non-mutually exclusive hypotheses that could explain the effect of the RdDM pathway on the triploid block. Through its canonical function, RdDM could affect DNA methylation at key loci, inducing the triploid block. Alternatively, NRPD1-dependent 21/22-nt siRNAs could mediate post-transcriptional gene silencing of key loci, consistent with the recently shown requirement of NRPD1 to produce 21/22-nt siRNAs [22, 23].

During pollen development, Pol IV generates an abundant class of 21/22-nt siRNAs (referred to as epigenetically activated siRNAs (easiRNAs)) that we previously proposed to act as the dosage-dependent signal inducing the triploid block [16, 44]. If Pol IV target sites remain methylated in the first generation of RdDM mutants (as shown for DMRi), they would still be able to recruit Pol IV, maintaining the production of easiRNAs and thus affect the triploid block. This could explain why loss of Pol IV function has a suppressive effect in the first homozygous generation, differing from *nrpe1* and *drm2* that required one additional generation to have an effect (Fig. 1). Once DNA methylation on Pol IV target sites is lost, which will happen after inbreeding of RdDM mutants, failure of Pol IV recruitment will abolish the signal and the triploid block is not triggered. We therefore propose that relevant DMRs establishing the triploid seed rescue are only erased after several rounds of inbreeding of RdDM mutants, thus corresponding to DMRi. These DMRs can then affect the transcription of surrounding genes/TEs or affect transcripts post-transcriptionally, resulting in the establishment of the triploid block.

In this and previous studies [16, 35], the *rdr2* mutant was found to have a substantially weaker effect compared to *nrpd1*. The function of Pol IV and RDR2 is generally coupled [45, 46]; it is therefore unexpected that the effect of mutants in *NRPD1* and *RDR2* differs. One plausible explanation could be the redundancy of RDR2 with RDR6 and RDR1 that all belong to the RDR alpha group [47]. While the functional roles of these three RDRs are generally distinct and their intracellular localization differs [48, 49], it is possible that during meiosis and the resulting breakdown of the nuclear envelope they can at least partially functionally substitute for each other. Similarly, also *dcl3* was previously found to have a weaker suppressive effect compared to *nrpd1* [16, 35], possibly caused by genetic redundancy of DCL3 with other DCLs [50, 51].

Interestingly, we found DMRi to overlap with genes that are potentially relevant for establishing the triploid block, like AGLs and ARFs [40]. Previous work from our group revealed that the AGL PHE1 is a central regulator of imprinted genes and loss of *PHE1* causes strong suppression of the triploid block [40]. We found a significant overlap of genes being negatively regulated upon loss of PHE1/PHE2 and NRPD1 function, suggesting a possible connection. Furthermore, ARFs overlapping with DMRi (*ARF12*, *13*, *14*, *20-23*) may mediate the response to the increased auxin level in triploid seeds that were previously shown to antagonize endosperm cellularization [52]. Whether increased dosage of easiRNAs negatively interferes with RdDM as previously proposed [16] remains to be further tested, but the strong overlap of loci marked by DMRi in the endosperm of triploid seeds suggests a possible connection. Successive inbreeding of *Arabidopsis ddm1* (*decrease in dna methylation1*) was shown to trigger enhanced phenotypic defects over generations caused by progressive loss of CG methylation as well as activation of alternative silencing mechanisms [53]. Similarly, *met1* mutants were shown to progressively form new and aberrant epigenetic patterns over generations [54]. In both mutants, a retargeting of H3K9me2 was observed, which depends on 21/22-nt easiRNAs [54, 55]. Whether the increase of CHH and CHG methylation upon loss of paternal NRPD1 function in the endosperm of triploid seeds is caused by a retargeting of H3K9me2 remains to be tested. It would provide an explanation for the finding that mutants homozygous for components of the RdDM pathway are able to suppress the triploid block [35].

In summary, in this study, we reveal that inbreeding of mutants impaired in RdDM components successively enhanced their ability to suppress the triploid block. Thus, loss of RdDM function differs in its effect in early and late generations, which has important implications when interpreting the effect of mutants impaired in RdDM function.

## Methods

### Plant growth and material

*Arabidopsis* mutants *nrpd1-3* (SALK_128428) [56], *nrpe1-12* (SALK_033852) [57], *rdr2-2* (SALK_059661) [58], and *drm2-2* (SALK_150863) [59] were obtained from Nottingham *Arabidopsis* Stock Centre (NASC). The *osd1-1* mutant [34] was kindly provided by Raphael Mercier. The tetraploid mutants *nrpd1-4* (SALK_083051), *nrpe1-11* (SALK_029919), *rdr2-1* (SAIL_1277_H08), *drm2-2*, and *dcl3-1* (SALK_005512) were kindly shared by Mary Gehring [35]. The used alleles are null alleles for the respective genes. The Col-0 accession was used as the wild type for all experiments. Primers used for genotyping all mutants are listed in Additional file 7: Table S6. *Arabidopsis* seeds were surface-sterilized in 5% commercial bleach and 0.01% Tween 20 for 10 min, followed by three times washes in sterile distilled, deionized water. Seeds were sown on half-strength Murashige and Skoog medium (0.43% [w/v] Murashige and Skoog salts, 0.8% [w/v] bacto agar, 0.19% [w/v] MES hydrate, and 1% [w/v] Suc). After stratification (2 days at 4°C), the plates were transferred to a growth chamber (16h of light/8h of dark, 110 μmol photons m^−2^ s^−1^, 21°C, 70% humidity). Ten-day-old seedlings were transferred to soil and grown in a growth chamber under a 16-h-light/8-h-dark cycle with a light intensity of 150 μmol photons m^−2^ s^−1^, at 21°C and 70% humidity.

For crosses, diploid Col-0 wild-type buds were emasculated 2 days before pollination with indicated pollen donors. One biological replicate corresponds to seeds of two to four crossed flowers from one inflorescence and plant.

### Bisulfite sequencing

*Arabidopsis* 3-week-old aerial parts were pooled from three plants as one replicate and ground with liquid nitrogen into fine powder and then used for isolation of genomic DNA using the MagJET Plant Genomic DNA Kit (K2761). Biological duplicates were generated for each genotype. Libraries were prepared with the Accel-NGS Methyl-Seq DNA Library Kit from Illumina (Cat No. 30096, Swift), and the sequencing was performed at Novogene (Hongkong, China) on a NovaSeq 6000 platform in 150-bp paired-end mode.

### Chromatin immunoprecipitation (ChIP) followed by sequencing

Cross-linked *Arabidopsis* leaves (100 mg) from each sample were ground in liquid nitrogen into fine powder and used for further experiments as previously described [37]. Biological triplicates were generated from each sample. Libraries were generated using 1.5 ng of starting material using the Ovation Ultralow Library System (NuGEN, San Carlos, USA), and the sequencing was performed at Novogene (Hongkong, China) on a HiSeqX in 150-bp paired-end mode. Anti-histone H3 (Sigma, #H9289) and anti-H3K9me2 (Diagenode, #pAb-060-050) antibodies were used in this study.

### Bioinformatic analysis

For DNA methylation analysis, 150-bp paired-end reads were trimmed by removing the first 5 bases from the 5′ end and the last 20 bases from the 3′ end. Reads were mapped to the *Arabidopsis* TAIR10 in paired-end mode (--score_min L,0,-0.6) genome using Bismark [60]. Duplicated reads were eliminated and methylation levels for each condition were calculated by averaging the two biological replicates (Additional file 1: Figure S7). Differentially methylated regions (DMRs) in CHG and CHH contexts were defined using 50-bp windows across the genome as units. Only hypomethylation in order wild type > F2 *nrpd1* (DMR1), F2 *nrpd1* > Fi *nrpd1* (DMRi), and wild type > Fi *nrpd1* (DMRx) was considered. Windows with differences in fractional methylation below the 1st decile (Fisher’s exact test *p*-value < 0.01) were selected and these were merged if they occurred within 300 bp (see Additional file 8: Table S7). Genes (gene-body plus 1kb upstream) overlapping with indicated DMRs were obtained using intersect feature of bedtools v2 [61]. TEs were assigned to family and super-family based on the current TAIR10 genome release.

ChIP-seq reads of three biological replicates passing a quality control were mapped to the *Arabidopsis* (TAIR10) genome using Bowtie [62] in single-end mode, allowing for up to two mismatches. Mapped reads were deduplicated and extended to the estimated average length of the genomic fragments (270 bp). Coverage was estimated and normalized to 10 million reads. H3K9me2 ChIP signals were normalized by subtracting their coverage with H3 ChIP data at every single position in the genome (Additional file 1: Figure S8).

For small RNA analysis, the resulting 18–30-bp-long sRNA reads after removing adapters were mapped to the *Arabidopsis* TAIR10 genome. After removing reads mapping to chloroplast and mitochondria and to structural noncoding RNAs (tRNAs, snRNAs, rRNAs, or snoRNAs), the resulting mapped reads from both replicates were pooled together, sorted in 21/22-nt and 24-nt categories, and remapped to the same reference masked genome mentioned above using ShorStack (–mismatches 0–mmap f) [63] in order to improve the localization of sRNAs mapping to defined DMR loci. The alignments were normalized by converting coverage values to RPM values.

PLAZA 4.0 dicots [64] was used to identify enriched Gene Ontology (GO) terms. GO terms of biological functions with *p*-value < 0.01 were further loaded on REVIGO [65] to remove the redundant terms. The charts were generated based on -log10 (*p*-values).

### Graphical and statistical software

To produce most graphs, base R functions were used. Hypergeometric, Wilcoxon, and ANOVA tests were performed in R. Screenshots of genes were exported using Integrated Genome Browser (IGB). Final figures were assembled using PowerPoint.

## Supplementary Information

**Additional file 1: Figure S1.** Rescue of 3x seed abortion using 4x RdDM mutants as pollen donor. **Figure S2.** Scheme of generating inbred RdDM *osd1* mutants and using them for crossing. **Figure S3.** Scheme of generating F2 and Fi *nrpd1-3* plants. **Figure S4.** Distribution of DMR1spec and DMRi over genomic features. **Figure S5.** Similar expression pattern of deregulated genes in 3x seeds and the endosperm of 3x seeds. **Figure S6.** Screenshots showed DNA methylation in flanking or coding regions of selected ARFs and AGLs. **Figure S7.** Correlation between replicates of CHG and CHH fractional methylation levels on Chr1. **Figure S8.** Correlation between three replicates of H3K9me2 - H3 methylation levels on Chr1 in wt leaves.

**Additional file 2: Table S1.** Quality of sequencing samples.

**Additional file 3: Table S2.** Lists of non-CG hypo DMRs.

**Additional file 4: Table S3.** List of DMRi overlapping deregulated genes in 3x seeds.

**Additional file 5: Table S4.** Genes with non-CG DMRi overlapping with non-CG hypo DMRs in endosperm of 3x seeds.

**Additional file 6: Table S5.** Genes losing and gaining non-CG methylation in endosperm of 3x and 3x *nrpd1* seeds.

**Additional file 7: Table S6.** Primer list.

**Additional file 8: Table S7.** Thresholds applied for DMRs.

**Additional file 9.** Review history.

## Data Availability

Publicly available datasets used in this study are as follows: methylation data in *Arabidopsis* RdDM mutants was from GSE39901 [38], small RNA data in *Arabidopsis* wild-type and *nrpd1* leaves was from [66], RNA-seq data of *Arabidopsis osd1 nrpd1* endosperm was from GSE84122 [16], RNA-seq of *Arabidopsis osd1 phe1 phe2* seeds was from GSE129744 [40], and DMRs and methylation data in the endosperm of *Arabidopsis* wt and *nrpd1* triploid seeds were from GSE126929 [35]. The sequencing data generated in this study are available in the Gene Expression Omnibus under accession number GSE156597 [67]. Additional file 2 Table S1 summarizes all sequencing data generated in this study.
