# Supplementary material for: Transgenerational effect of mutants in the RNA-directed DNA methylation pathway on the triploid block in Arabidopsis
Source: Genome Biol. 2021 May 6;22:141. doi: 10.1186/s13059-021-02359-2 (PMC8101200; doi:10.1186/s13059-021-02359-2)
Supplement: Supplementary file 1 — Additional file 1: Figure S1. Rescue of 3x seed abortion using 4x RdDM mutants as pollen donor. Figure S2. Scheme of generating inbred RdDM osd1 mutants and using them for crossing. Figure S3. Scheme of generating F2 and Fi nrpd1-3 plants. Figure S4. Distribution of DMR1spec and DMRi over genomic features. Figure S5. Similar expression pattern of deregulated genes in 3x seeds and the endosperm of 3x seeds. Figure S6. Screenshots showed DNA methylation in flanking or coding regions of selected ARFs and AGLs. Figure S7. Correlation between replicates of CHG and CHH fractional methylation levels on Chr1. Figure S8. Correlation between three replicates of H3K9me2 - H3 methylation levels on Chr1 in wt leaves. [file 13059_2021_2359_MOESM1_ESM.pdf]

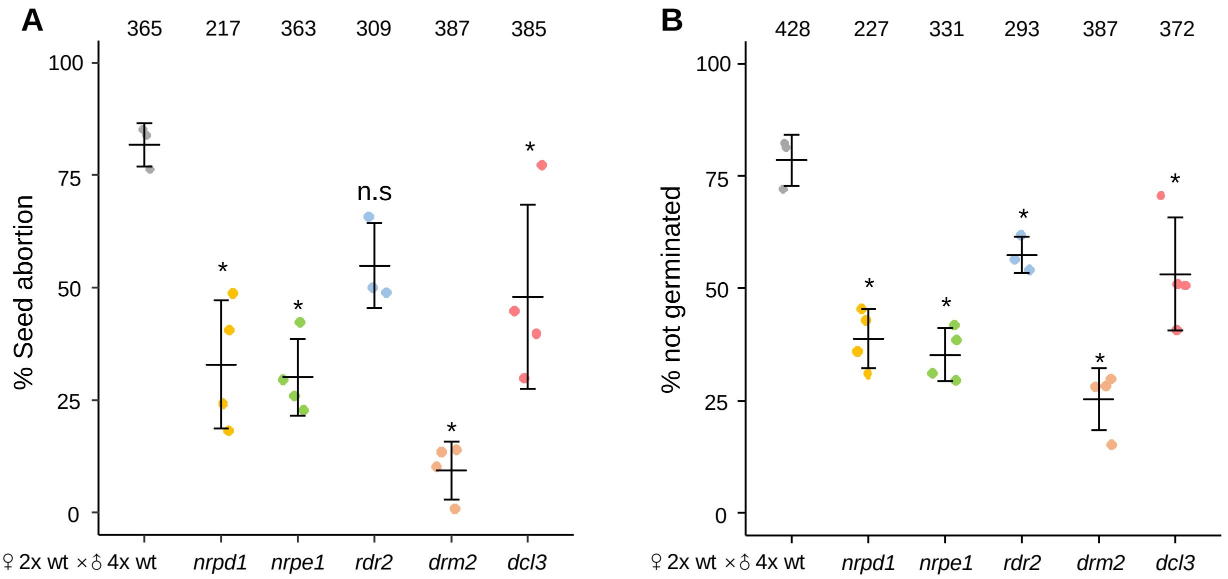

Figure S1. Rescue of 3x seed abortion using 4x RdDM mutants as pollen donor.
(A) Each dot represents the seed abortion rate of 2-4 siliques from a single inflorescence pooled as one cross.
(B) Each dot represents the percentage of seeds that failed to germinate from each cross.
Bars show means and the range of standard deviation. Numbers on top represent total seed numbers.
* represents statistically significant difference ($P < 0.05$) in comparisons between the indicated cross using 4x RdDM mutants and the cross using 4x wild type as pollen donor. n.s, not significant. Statistical significance calculated by ANOVA with post-hoc Tukey HSD Test.

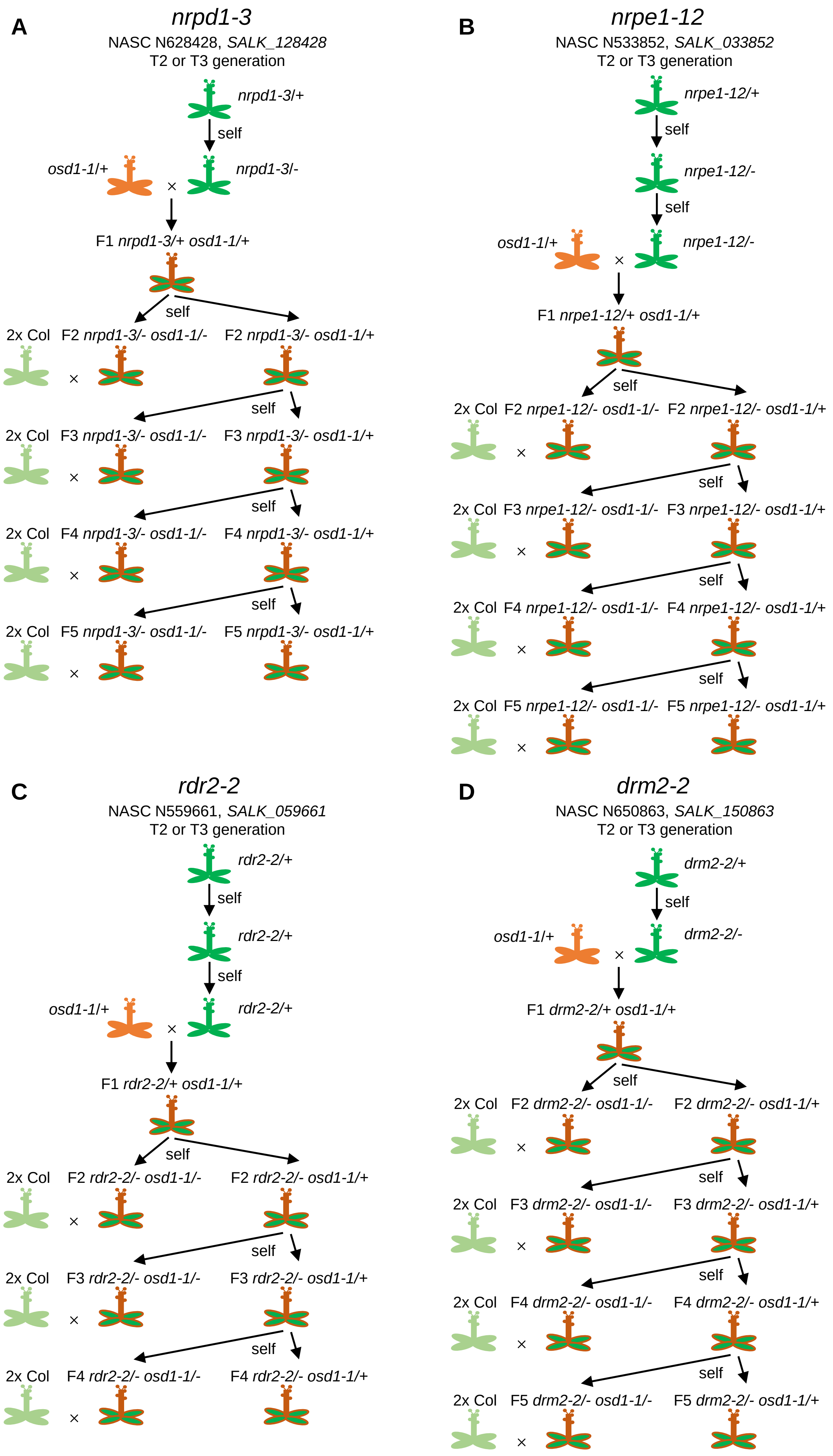

Figure S2. Scheme of generating inbred RdDM *osd1* mutants and using them for crossing.
(A) *nrpd1-3*, **(B)** *nrpe1-12*, **(C)** *rdr2-2*, and **(D)** *drm2-2*. All mutants were obtained from the Nottingham Arabidopsis Stock Centre (NASC) as T2 or T3 generation according to the supplied information, propagated for one or two generations in lab, and then crossed with *osd1-1* heterozygote to generate RdDM *osd1* mutants for subsequent tests. Diploid wild-type plants (2x Col) were pollinated using RdDM *osd1* double homozygous plants identified at each generation as father.

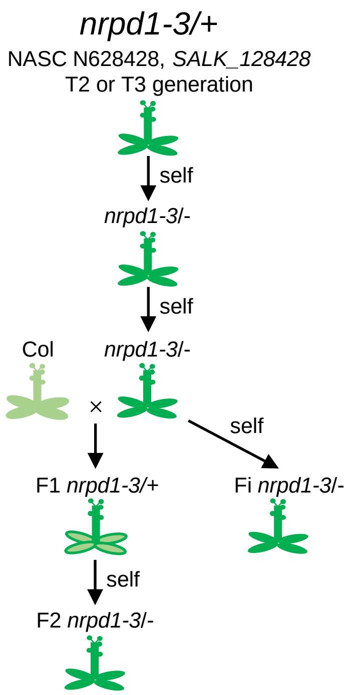

Figure S3. Scheme of generating F2 and Fi *nrpd1-3* plants. The *nrpd1-3* mutant seeds, T2 or T3 generation, were obtained from the Nottingham Arabidopsis Stock Centre (NASC), propagated for two generations in lab, and then crossed with wild type (Col) to generate *nrpd1-3* heterozygote. First homozygous *nrpd1-3* plants were genotyped at F2 generation. The inbred *nrpd1-3* homozygous plants (Fi) were propagated for three generations in lab.

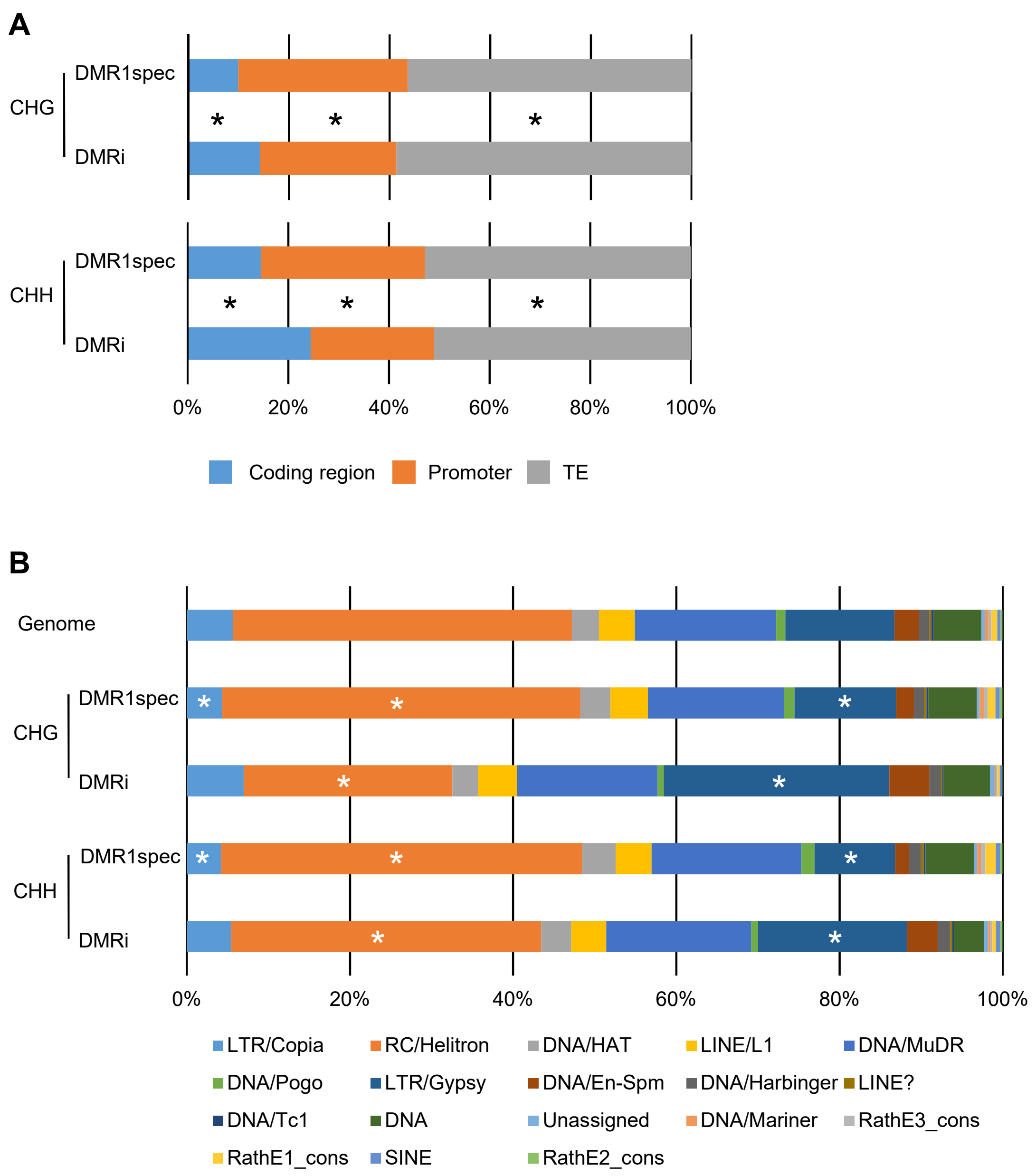

Figure S4. Distribution of DMR1spec and DMRi over genomic features.
(A) Distribution of non-CG DMR1spec and DMRi over genomic features. Asterisks denote significant differences between DMR1spec and DMRi ($P < 0.001$; Chi square test).
(B) Percentage of TE families intersected with non-CG DMR1spec and DMRi. Asterisks denote significant differences between observed and expected enrichments of TE families ($P < 0.001$; Chi square test).

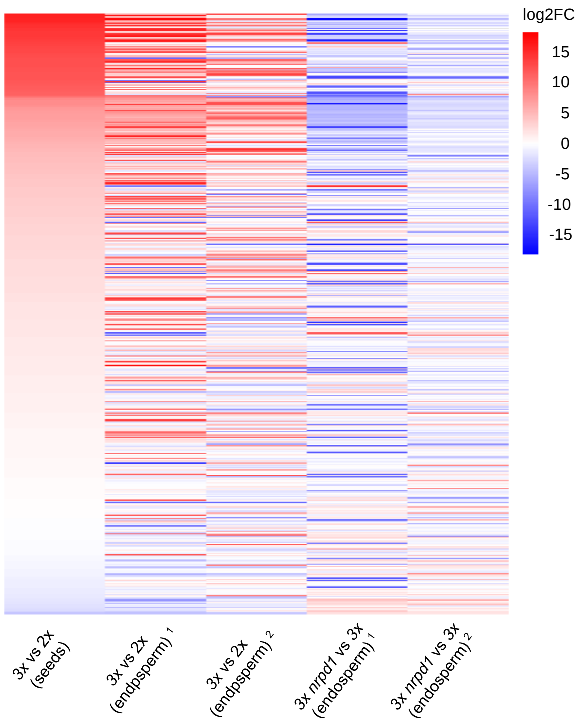

Figure S5. Similar expression pattern of deregulated genes in 3x seeds and the endosperm of 3x seeds.
Heatmap showing gene expression of downregulated genes (log2FC < -1, $p < 0.05$) in 3x *phe1 phe2* vs 3x seeds [41] and their expression in 3x vs 2x seeds [41] and two sources of endosperm of 3x vs 2x seeds and endosperm of 3x *nrpd1* vs 3x seeds. Dataset 1 [16] and dataset 2 [35].

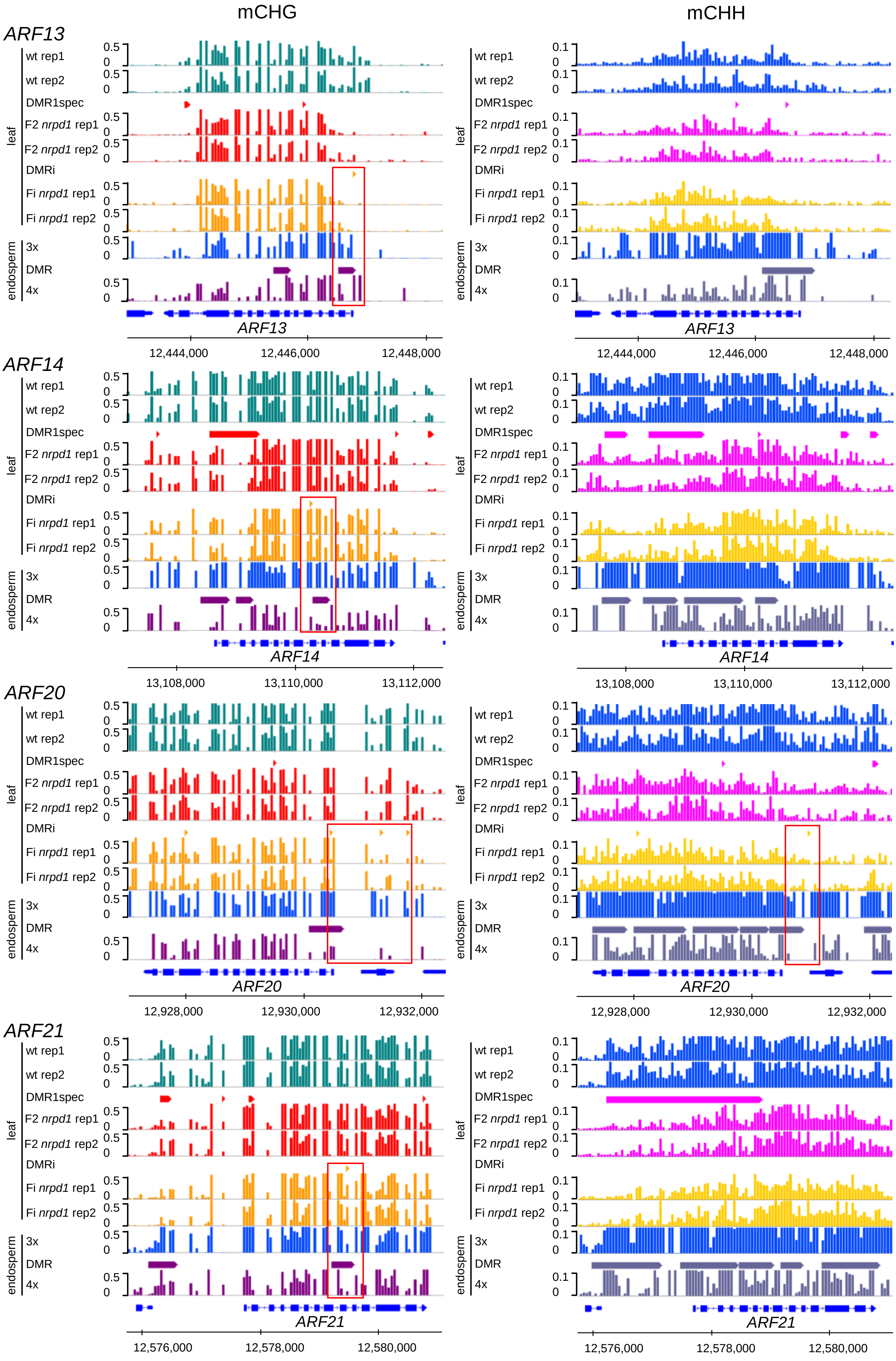

mCHG
mCHH
ARF13
ARF14
ARF20
ARF21
wt rep1
wt rep2
DMR1spec
F2 nrpd1 rep1
F2 nrpd1 rep2
DMRi
Fi nrpd1 rep1
Fi nrpd1 rep2
3x
DMR
4x
leaf
endosperm
12,444,000
12,446,000
12,448,000
13,108,000
13,110,000
13,112,000
12,928,000
12,930,000
12,932,000
12,576,000
12,578,000
12,580,000

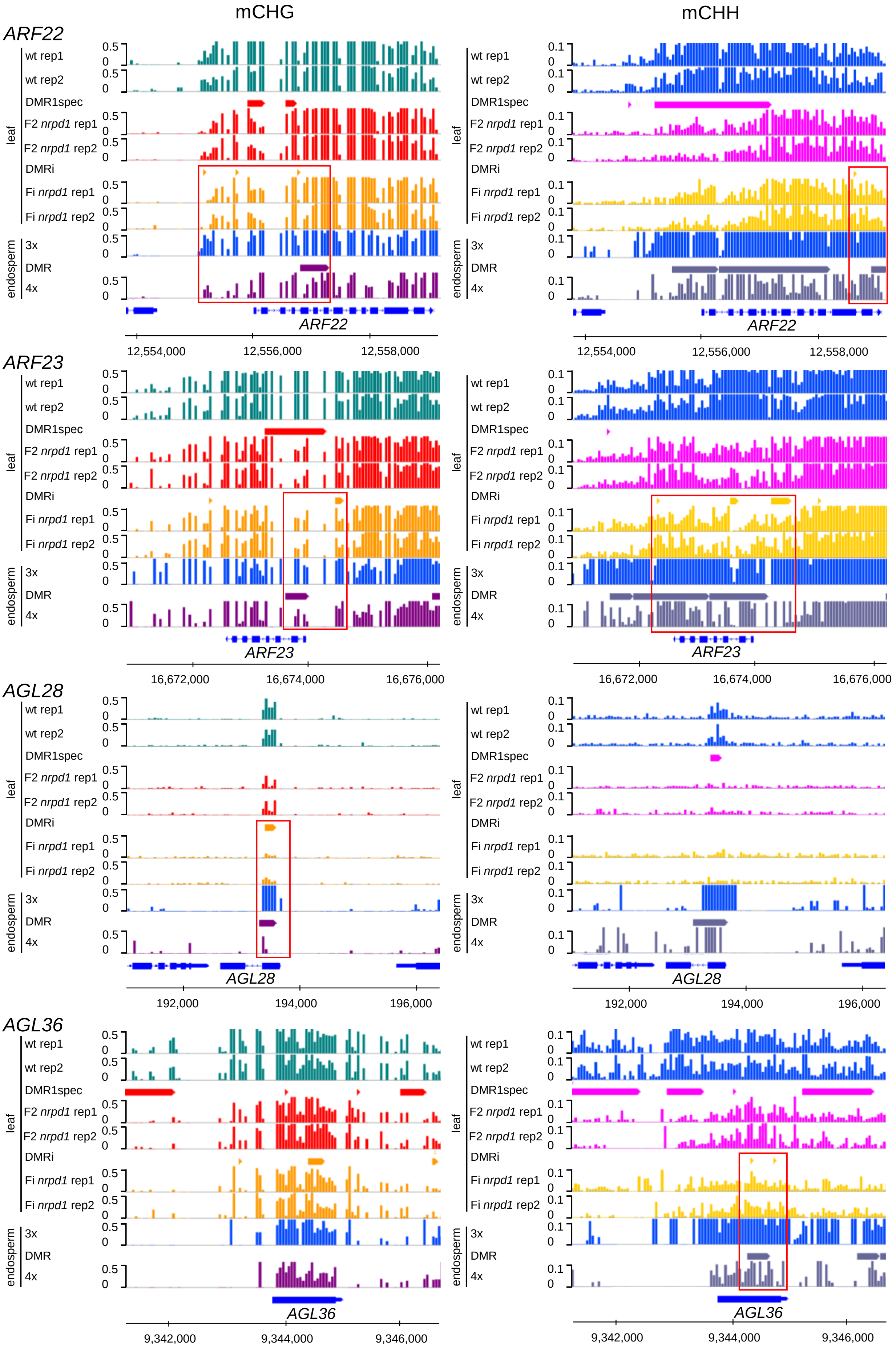

Figure S6. Screenshots showed DNA methylation in flanking or coding regions of selected ARFs and AGLs. *ARFs* and *AGLs* losing non-CG methylation during generations in *nrpd1* leaves and endosperm of 3x seeds [35]. Bars on top represent DMRs. Red boxes highlight regions with DMRi. Endosperm in 2x seeds is triploid (3x) and tetraploid (4x) in 3x seeds.

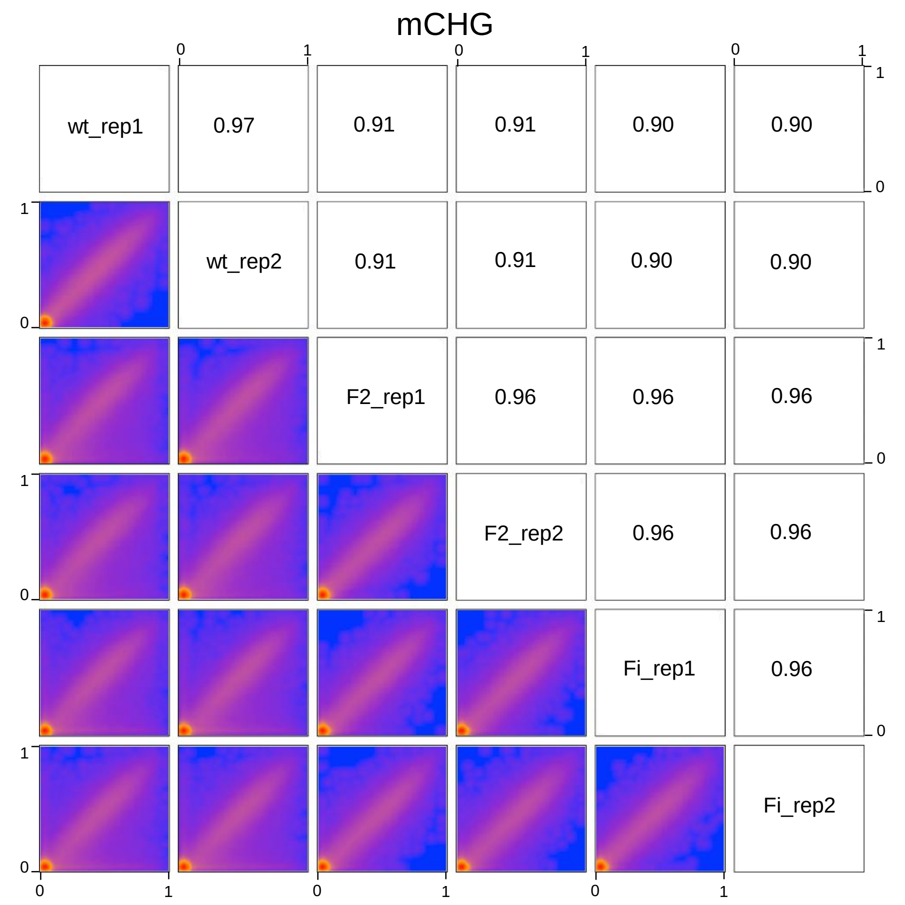

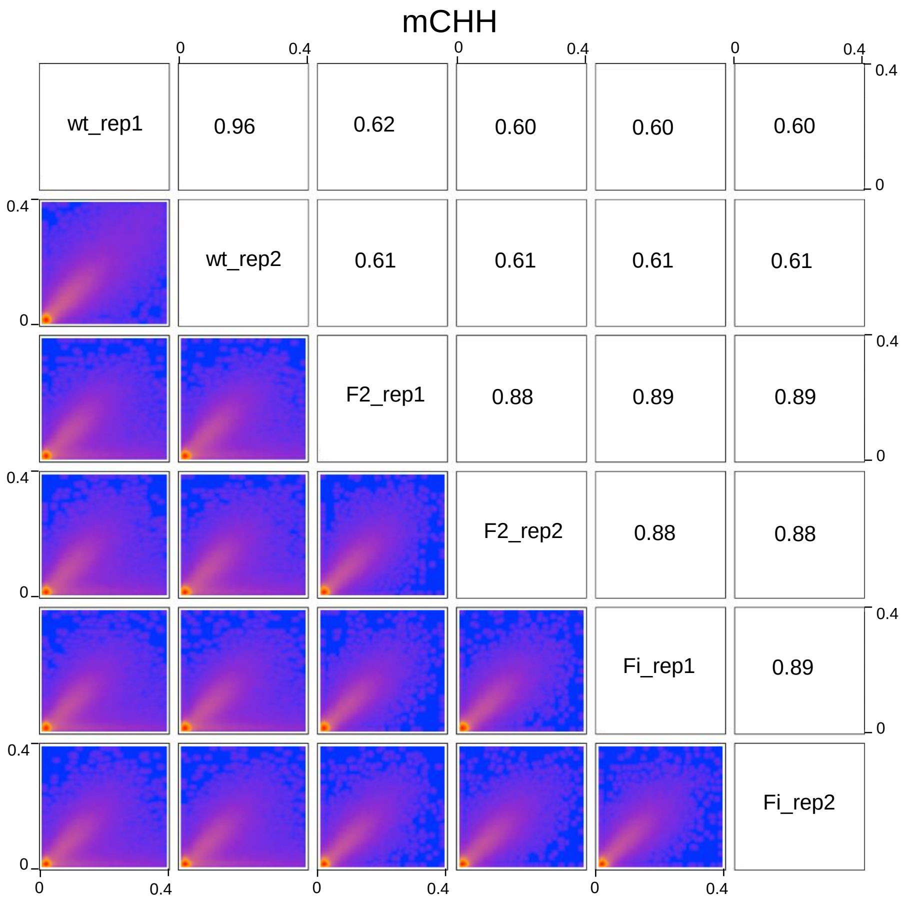

Figure S7. Correlation between replicates of CHG and CHH fractional methylation levels on Chr1. Two biological replicates of wt, F2 *nrpd1*, and Fi *nrpd1* plants were present. Lower diagonal panel shows smoothed color density scatterplots of average methylation levels in 50 bp bins between replicates. Upper diagonal panel shows Pearson correlation coefficients.

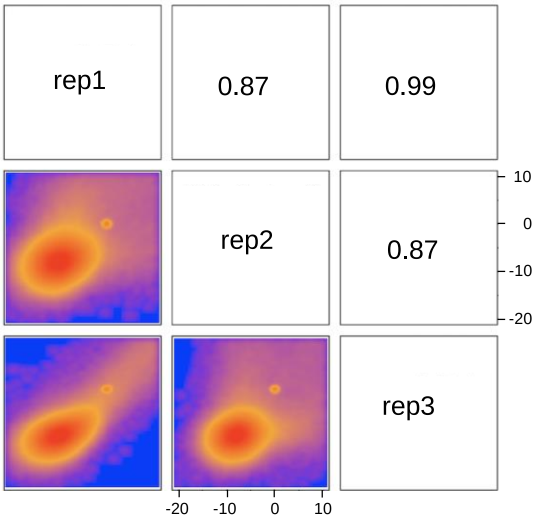

Figure S8. Correlation between three replicates of H3k9m2 - H3 methylation levels on Chr1 in wt leaves. Lower diagonal panel shows smoothed color density of average methylation level in 50 bp bins. Upper diagonal panel shows Pearson correlation coefficients.
