# Supplementary material for: Transgenerational effect of mutants in the RNA-directed DNA methylation pathway on the triploid block in Arabidopsis
Source: Genome Biol. 2021 May 6;22:141. doi: 10.1186/s13059-021-02359-2 (PMC8101200; doi:10.1186/s13059-021-02359-2)
Supplement: Supplementary file 2 — Additional file 2: Table S1. Quality of sequencing samples. [file 13059_2021_2359_MOESM2_ESM.docx]

| ChIP-seq | N of total reads | N of reads mapped | Mapping efficiency % | N of reads after deduplication |  |  |
| --- | --- | --- | --- | --- | --- | --- |
| H3_wt_repliate1 | 26 784 940 | 21 383 156 | 79.8 | 3 843 943 |  |  |
| H3_wt_repliate2 | 26 976 684 | 21 562 914 | 79.9 | 3 416 121 |  |  |
| H3_wt_repliate3 | 16 335 379 | 13 145 064 | 80.5 | 1 912 000 |  |  |
| H3K9me2_wt_repliate1 | 26 479 772 | 18 974 705 | 71.7 | 5 113 476 |  |  |
| H3K9me2_wt_repliate2 | 12 228 351 | 9 896 048 | 80.9 | 1 867 201 |  |  |
| H3K9me2_wt_repliate3 | 28 051 355 | 21 467 034 | 76.5 | 6 225 712 |  |  |
|  | | | | | | |
| Bisulfite-seq | **N of trimmed reads** | **N of mapped reads** | **Mapping efficiency% %%** | **Genome**  **Coverage** | **conversion rates %** |  |
| wt_replicate1 | 56 020 045 | 44 428 200 | 79.3 | 71.7 | 99.06 |  |
| wt_replicate1 | 54 051 345 | 42 866 582 | 79.3 | 85.8 | 99.05 |  |
| F2*_nrpd1_*replicate1 | 51 954 677 | 38 694 675 | 74.5 | 77.5 | 99.05 |  |
| F2*_nrpd1_*replicate2 | 48 802 224 | 35 921 742 | 73.6 | 71.9 | 99.08 |  |
| Fi*_nrpd1_*replicate1 | 49 943 082 | 37 827 026 | 75.7 | 75.7 | 99.01 |  |
| Fi*_nrpd1_*replicate2 | 53 643 465 | 40 017 936 | 74.6 | 80.1 | 99.15 |  |

**Table S1. Quality of sequencing samples.**

Table shows details of the sequenced samples generated in this study. Replicates are biological replicates.
