# Supplementary material for: Transgenerational effect of mutants in the RNA-directed DNA methylation pathway on the triploid block in Arabidopsis
Source: Genome Biol. 2021 May 6;22:141. doi: 10.1186/s13059-021-02359-2 (PMC8101200; doi:10.1186/s13059-021-02359-2)
Supplement: Supplementary file 7 — Additional file 7: Table S6. Primer list. [file 13059_2021_2359_MOESM7_ESM.docx]

**Table S6. Primer list**

| Primer names | Sequences | Application |
| --- | --- | --- |
| *nrpd1-3*_LP | GATCTGTTCAGCTTGCTCGTC | PCR for identifying *nrpd1-3* mutation |
| *nrpd1-3*_RP | TTAATGTTCTTCATGCGGGAC | PCR for identifying *nrpd1-3* mutation |
| *nrpe1-12*_LP | GCTTTGACCCGATCCTTAAAC | PCR for identifying *nrpe1-12* mutation |
| *nrpe1-12*_RP | TTATTTTTGTCCCTGGAACCC | PCR for identifying *nrpe1-12* mutation |
| *rdr2-2*_LP | CTGATCGCGAGATTTCAGTTC | PCR for identifying *rdr2-2* mutation |
| *rdr2-2*_RP | AGAAGATTGGAGCAAGCTTCC | PCR for identifying *rdr2-2* mutation |
| *drm2-2*_LP | AGATCGCTTCCAGAGTTAGCC | PCR for identifying *drm2-2* mutation |
| *drm2-2*_RP | TTGTCGCAAAAAGCAAAAGAG | PCR for identifying *drm2-2* mutation |
| *nrpd1-4*_LP | TGGGTTTGCCATTTTCATATC | PCR for identifying *nrpd1-4* mutation |
| *nrpd1-4*_RP | GCATGCTTGAGTAAAAGGTGC | PCR for identifying *nrpd1-4* mutation |
| *nrpe1-11*_LP | ATTTCTTCTTTGATGGGGGAG | PCR for identifying *nrpe1-11* mutation |
| *nrpe1-11*_RP | TGTCGTGGATATGACCATTTG | PCR for identifying *nrpe1-11* mutation |
| *rdr2-1*_LP | GGCAATAAGAGTGATTGTGCC | PCR for identifying *rdr2-1* mutation |
| *rdr2-1*_RP | TCTCATGCACGTTCATCAAAC | PCR for identifying *rdr2-1* mutation |
| *dcl3-1_LP* | ACAGGTAACCTTGCCATGTTG | PCR for identifying *dcl3-1* mutation |
| *dcl3-1_RP* | TGGAAAAGTTTGCTACAACGG | PCR for identifying *dcl3-1* mutation |
| LBb1.3 | ATTTTGCCGATTTCGGAAC | SALK line T-DNA primer |
| LB1 | GCCTTTTCAGAAATGGATAAATAGCCTTGCTTCC | SAIL line T-DNA primer |
