# Supplementary material for: Transgenerational effect of mutants in the RNA-directed DNA methylation pathway on the triploid block in Arabidopsis
Source: Genome Biol. 2021 May 6;22:141. doi: 10.1186/s13059-021-02359-2 (PMC8101200; doi:10.1186/s13059-021-02359-2)
Supplement: Supplementary file 8 — Additional file 8: Table S7. Thresholds applied for DMRs. [file 13059_2021_2359_MOESM8_ESM.docx]

**Table S7. Thresholds applied for DMRs**

| DMR_name_manus | fractional methylation threshold (below the 1st decile) | n of DMRs (single50 bp bins) | n of DMRs (merged when closer than 300 bp) |
| --- | --- | --- | --- |
| DMRi CHG | 0.02564103 | 16 487 | 10 327 |
| DMRi CHH | 0.00853772 | 16 313 | 12 819 |
| DMR1 CHG | 0.02199014 | 38 588 | 12 652 |
| DMR1 CHH | 0.01215805 | 102 524 | 18 497 |
| DMRx CHG | 0.02825534 | 51 583 | 15 132 |
| DMRx CHH | 0.01339286 | 111 176 | 18 781 |
