# Supplementary material for: Transgenerational effect of mutants in the RNA-directed DNA methylation pathway on the triploid block in Arabidopsis
Source: Genome Biol. 2021 May 6;22:141. doi: 10.1186/s13059-021-02359-2 (PMC8101200; doi:10.1186/s13059-021-02359-2)
Supplement: Supplementary file 9 — Additional file 9. Review history. [file 13059_2021_2359_MOESM9_ESM.docx]

Review History

**First round of review**

**Reviewer 1**

**Are you able to assess all statistics in the manuscript, including the appropriateness of statistical tests used?** Yes, and I have assessed the statistics in my report.

**Comments to author:**

In the current work, Wang et al. investigate partly conflicting reports regarding the ability of RdDM pathway mutants to suppress the triploid block phenomenon in Arabidopsis thaliana.  The triploid block leads to seed abortion when paternal and maternal contributions are out of balance in the plant endosperm. The endosperm is normally triploid (3n), which results from fertilization of the diploid (2n) central cell in the female gametophyte with a haploid (1n) sperm cell from the pollen.  Previous work in the Kohler lab studied the connections between the RdDM pathway and the triploid block phenomena by generating 2n sperm cells using pollen from the osd1 mutant and found only osd1nrpd1 mutants suppress the triploid block and generate viable seeds. Work from the Gehring lab generated 2n sperm cells by generating tetraploid plants using a chemical approach (colchicine treatment) and found many different RdDM mutants suppress the triploid block.  Based on these results, it remained unclear if the canonical role of the RdDM pathway in targeting DNA methylation was required to suppress the triploid block or if another role, independent of the rest of the RdDM factors, was responsible.  Here, the authors present work showing that upon inbreeding several osd1 mutant combinations (i.e. osd1nrpe1and osd1drm2) are now able to suppress the triploid block whereas they did not in the first generation. They then hypothesize that this difference could be due to transgenerational differences in DNA methylation patterns. Indeed, they show that the nrpd1 mutant initially (1st generation) shows losses of non-CG methylation at many sites "DMR1"and that after several generations of inbreeding there are additional sites "DMRi" that show a more gradual decreases in non-CG methylation.   Mechanistically, they present data on the locations, types of transposons, and genetic pathways controlling methylation at DMR1 vs DMRi sites and conclude that sites that gradually lose methylation (DMRi) rely on both RdDM and CMT pathways. Finally, the authors look at the overlaps between DMRi locations and genes mis-regulated in connection with the triploid block.  Here, they find an enrichment in genes related to transcriptional regulation and auxin signaling.  However, it is not clear what the mechanism would be, as these regions show only modest decreases in methylation in DMRi vs DMR1 (at least in leaves) and are correlated with the downregulation of expression in nrpd1 rather than the release of silencing (in endosperm). Although such mechanistic questions remain, this work provides clarity on the roles of the RdDM pathway in suppressing the triploid block, characterizes the behaviors of loci that lose methylation immediately or gradually upon disruption of the RdDM pathway, and provides testable hypotheses about the connections between DNA methylation, auxin signaling and the triploid block.  


Main comments: Here, I have three main comments, each related to aspects central to the work presented.  The first asks for clarification on the genetic materials used to assess the effect of inbreeding on suppression of the triploid block (see 1. Genetic Materials), the second relates to the connections between DNA methylation changes at genes implicated in the triploid block (see 2. DMRs) and the third pertains to the apparent disconnect between the role of RdDM in gene silencing and the downregulation of genes associated with hypo DMRs in nrpd1 mutants (see 3. Model).  

1.     Genetic Materials: Figure 1 nicely demonstrates that upon inbreeding the osd1nprd1, osd1nrpe1, and osd1drm2 lines are all able to suppress the triploid block.  However, in Fig S2, which outlines the approach for generating these inbred lines, it is not clear what the genotypes of the starting mutants were.  For example, were the nrpd1, nrpe1, rdr2, and drm2 mutant keep heterozygous prior to the initial cross to the osd1 mutant?  If not, when were they taken to homozygosity? This is an important point as recent studies have show that there are some sites in nprd1-/- mutants where methylation cannot be restored by adding back a functional NRPD1 gene (PMID: 32514141), demonstrating that the past history of these lines could be critical. As is done for the bottom portions of the graphics, please indicated the genotypes of the mutants selected for the initial selfing and the first cross to the osd1 mutant.

2.     DMRs: To better evaluated the potential significance of the DMRs from leaves extrapolated onto data from the endosperm, additional information about the data processing and analyses are required. Ideally, one would directly test the effects of methylation at these loci over several generations directly in endosperm, but given the difficulties and costs of such assays, it may be outside the scope of the current work.  Nonetheless, additional information about the current data could already be helpful assessing the reproducibility and scale of methylation differences observed at these loci in leaf tissue upon inbreeding.  
*     Replicate Data: Based on the coverage (Table S1) the WT, F2 nrpd1, and Fi nrpd1 were all sufficiently high to treat independently. Are the DMRs presented in Table S2 those that were identified between both WT replicates and either both F2 nrpd1 or both Fi nrpd1 replicates indicating the most reproducible DMRs or are they all the pair-wise DMRs?  For bulk analyses, like Fig. 2G or Fig. S4, including all the pairwise vs the more robust DMRs would likely not make much difference, but for the regions highlighted in Fig. 6B and Fig. S6 it would very informative to know how reproducible these DMRs are. Along these same lines, given the scale of the screen shots shown, its difficult to evaluate the changes in methylation at these DMRs.  I would suggest showing a zoomed in view and labeling the regions with the % methylation in each genotype and displaying the two biological replicates separately.
*     DMR Thresholds: Fig 2F, it seems that DMRs showing very little changes in methylation were included (see bottom 1/5th of the heatmaps) which could introduce a large about of noise in the regions being studied.  The inclusion of DMRs that show minimal differences could also affect the overall conclusions of the study, if for example, the DMRs highlighted in Fig. 6B and Fig. S6 all fall into this category.  Its not clear from the methods how this threshold was set and if it also has a % methylation change cutoff or just a p-value cutoff.  Please clarify the methods and indicate where the DMRs in Fig. 6B and Fig. S6 fall on these heatmaps.

3.     Model:  
*     While the RdDM pathway is quite complicated, as are the various other pathways and proteins influencing the triploid block, the fact that genes associated with losses of methylation in nrpd1 mutants are downregulated in the endosperm seems at odds with current models of gene silencing.  Do the authors have a model that would explain this disconnect that could be presented in the discussion?
*     Also the fact that rdr2 mutants fail to suppress the triploid block is at odds most RdDM models (as mentioned in the discussion) and suggest perhaps Pol-IV and another RDR are required to make siRNAs that rather than targeting methylation participated in post-transcriptional gene silencing.  In this case, loss of the downstream RdDM machinery would suppress the triploid block not because of alterations in methylation that affect gene regulation at the transcriptional level, but rather alterations in methylation that are required for Pol-IV targeting. This is alluded to in the discussion, but is not directly presented as an alternative hypothesis to their model wherein losses of methylation lead to the downregulation of specific target genes. Given the lack of transgenerational methylation data in nrpd1 and nrpe1/drm2 endosperm, I think both models should be presented.


Minor comments
*     I would suggest adding Table S7 to Table S2.  Also, the numbers of DMRs in the Venn diagrams (Fig. 2) don't match perfectly the numbers in Table S2/S7.  Please check and correct as needed.  
*     I found the data presentation in Fig S4A,B was more intuitive than in Fig 4A,B.  As they are showing the same data, just plotted different ways, and take similar space, I would suggest moving the boxplots from Fig. S4 to the main figure.
*     Please add information to the methods about how the biological replicates for the ChIPseq data were taken into account for generating the boxplots in Fig. 4C, D.
*     Please cite in the main text sources of genomic data re-analyzed for this study.  For example, in the section covering the transcriptomic data in the endosperm and seeds it is not clear this was previously published data.
*     In the main text, ln 230, endosperm of 3x vs 2x seeds are mentioned but in Fig. 6B and Fig. S6 the endosperm tracks are labeled as 3x and 4x.  Please clarify.
*     Please add a description of the color bar for Fig. 2F.  I assume it is showing % methylation for each DMR?
*     Ln 59  "stablished" should be "established"
*     Ln 71-72  What is meant by "paternally-biased"?
*     Ln 76  Should "eukaryotic TE" be "euchromatic TE"?

**Reviewer 2**

**Are you able to assess all statistics in the manuscript, including the appropriateness of statistical tests used?** No, I do not feel adequately qualified to assess the statistics.

**Comments to author:**

The manuscript by Wang et al. addresses the molecular mechanisms responsible for triploid block in plants. This phenomenon attracted a lot of recent attention and has been shown to be controlled by RNA silencing. Two groups reported conflicting results about the involvement of particular silencing factors. These inconsistent results greatly affected the ability to interpret existing data. This manuscript resolves this inconsistency and shows that it is caused by a factor previously assumed to be irrelevant - inbreeding of RdDM mutants. This is an important and timely discovery. The manuscript further investigates the impact of inbreeding on accumulating changes in DNA methylation and expression of relevant genes. This aspect of the manuscript combines high quality results with insights that are less complete.
Under more typical circumstances, the preliminary nature of some of the presented work would justify major criticism. In this case, it should however be balanced by the timeliness of this study. The conflicting data have been published less than two years ago and resolving the existing conundrum required performing lengthy inbreeding crosses. The field will greatly benefit from this study being published as soon as possible.

Specific comments
1. DNA methylation has been assayed mostly in leaves while triploid block occurs in developing seeds. The limitations of this approach should at least be openly addressed in the manuscript.
2. The vaguely explained Fi population (more than three times inbred) is not optimal but still acceptable given the time needed to perform inbreeding. It should however be explained in as much detail as possible.
3. Classification of DMRs shown in Fig. 3 offers very little insights into the studied process and could be moved to the supplement (or removed from the manuscript).
4. The observation that loci that progressively lose DNA methylation depend on CMT2 and CMT3 is very interesting but not much is offered in terms of explaining why CMT2 and CMT3 cannot maintain silencing in the absence of RdDM. Addressing this question directly may be difficult without lengthy inbreeding experiments with DNA methyltransferase mutants, however any additional insights based on existing datasets would strengthen the manuscript. A thorough discussion of this issue is needed.
5. Data shown in Fig. 5A do not convince that CHH methylation has no impact on expression of relevant genes. A more in depth analysis not based on multiple arbitrary thresholds would be needed to support this conclusion.
6. One label on Fig. 5A is not clear "Genes upregulated in endosperm of 3x and downregulated in 3x nrpd1 seeds". Did the authors mean "… or downregulated…"?
7. GO analysis shown in Fig. 5B is not particularly conclusive and should be interpreted carefully.
8. The effects of accumulating changes in DNA methylation on genes that encode proteins, which directly affect endosperm development, although addressed in the manuscript, are not clear. The ability to attribute the observed effects to particular target genes would certainly strengthen this study but may require extensive additional experimental effort. A more direct explanation of the limitations of the obtained insights would help the readers better understand this aspect of the manuscript.
9. The last section of the results is very confusing. It appears to address an important issue of DNA methylation in leaves vs. in seeds. However - as explained above - this issue is not properly explained, and this section comes an unpleasant surprise. It should be reworded and better integrated with the rest of the manuscript.
10. The results of this study are likely to be relevant beyond the process of triploid block and the manuscript would greatly benefit from putting the obtained results in context of earlier studies where accumulating defects were observed in met1 and ddm1 mutants but not in RdDM mutants.
11. It is not clear how duplicated (WGBS) or triplicated (ChIP-seq) datasets were analyzed to demonstrate reproducibility.

**Reviewer 3**

**Are you able to assess all statistics in the manuscript, including the appropriateness of statistical tests used?** Yes, and I have assessed the statistics in my report.

**Comments to author:**

This submission describes a genetic and molecular analysis of triploid block suppression across multiple generations of inbreeding of several Arabidopsis RdDM mutants.  DNA methylation is also analyzed in multiple genotypes across generations.  The methods are appropriate and well described, including suitable controls. The work is important in understanding some previously reported discrepancies with regard to the genetic requirement of some factors in suppressing the triploid block.  The authors reasonably assert that the differences previously observed are related to the level of inbreeding in the RdDM backgrounds, which differed between the two studies.  This assertion is tested adequately with this work, and the results are fairly interpreted.  This work is likely to be of broad interest to many in the field of gene silencing and provides insight into the role of epigenetic regulation in detection and respond to ploidy during early development.

I have three comments that are worthy of consideration but do not substantially change my opinion of the work.  

First, although the overall crossing strategy is clearly described in the supplement figure, it is not clear if the starting stocks were heterozygous or homozygous insertion lines, which might mean that the individuals crossed with osd1 had experienced homozygosity of the RdDM mutations for differing numbers of generations.  This may or may not change anything about the interpretation of the reported results, but give the apparent importance of inbreeding, it would be useful to describe this clearly.  Also, although it may be obvious for scientists very close to this work, it would be nice to state if all of the mutants utilized contain null mutations for the genes of interest.

Second, with regard to the data reported in Figure 1 for seed germination and abortion, it is not completely clear if the two panels of data represent mutually exclusive seed populations.  In other words, is it % germination of all seed (including normal and aborted) or of normal seed only?  Also, n (number of seed for each analysis) is not indicated for any of this data (aside from a note about the number of siliques constituting a biological replicate).

Thirdly, related to the second comment, the figure legends are not very detailed and are missing some potentially useful information.  Including information about what data is actually plotted, all acronyms used should be explained in figure legends but in many cases are not.  For example, in the legend for figure 2, it is stated that DMRs is an acronym for differentially methylated regions; the use of an 's' in the acronym here is a little confusing given the very specifically defined group of differentially methylated regions designated as DMR1s in the results section.  DMR1s and other designations are also used in Figure 3, without any explanation in the legends.  I also do not see much information about how the TEs analysis was completed, or at least a citation for the definition of the different families.

**Authors Response**

**We would like to thank all three reviewers for their constructive comments aiming to improve our manuscript.**

Reviewer #1:

In the current work, Wang et al. investigate partly conflicting reports regarding the ability of RdDM pathway mutants to suppress the triploid block phenomenon in Arabidopsis thaliana.  The triploid block leads to seed abortion when paternal and maternal contributions are out of balance in the plant endosperm. The endosperm is normally triploid (3n), which results from fertilization of the diploid (2n) central cell in the female gametophyte with a haploid (1n) sperm cell from the pollen.  Previous work in the Kohler lab studied the connections between the RdDM pathway and the triploid block phenomena by generating 2n sperm cells using pollen from the osd1 mutant and found only osd1nrpd1 mutants suppress the triploid block and generate viable seeds. Work from the Gehring lab generated 2n sperm cells by generating tetraploid plants using a chemical approach (colchicine treatment) and found many different RdDM mutants suppress the triploid block.  Based on these results, it remained unclear if the canonical role of the RdDM pathway in targeting DNA methylation was required to suppress the triploid block or if another role, independent of the rest of the RdDM factors, was responsible.  Here, the authors present work showing that upon inbreeding several osd1 mutant combinations (i.e. osd1nrpe1and osd1drm2) are now able to suppress the triploid block whereas they did not in the first generation. They then hypothesize that this difference could be due to transgenerational differences in DNA methylation patterns. Indeed, they show that the nrpd1 mutant initially (1st generation) shows losses of non-CG methylation at many sites "DMR1"and that after several generations of inbreeding there are additional sites "DMRi" that show a more gradual decreases in non-CG methylation.   Mechanistically, they present data on the locations, types of transposons, and genetic pathways controlling methylation at DMR1 vs DMRi sites and conclude that sites

that gradually lose methylation (DMRi) rely on both RdDM and CMT pathways. Finally, the authors look at the overlaps between DMRi locations and genes mis-regulated in connection with the triploid block.  Here, they find an enrichment in genes related to transcriptional regulation and auxin signaling.  However, it is not clear what the mechanism would be, as these regions show only modest decreases in methylation in DMRi vs DMR1 (at least in leaves) and are correlated with the downregulation of expression in nrpd1 rather than the release of silencing (in endosperm). Although such mechanistic questions remain, this work provides clarity on the roles of the RdDM pathway in suppressing the triploid block, characterizes the behaviors of loci that lose methylation immediately or gradually upon disruption of the RdDM pathway, and provides testable hypotheses about the connections between DNA methylation, auxin signaling and the triploid block.

Main comments: Here, I have three main comments, each related to aspects central to the work presented.  The first asks for clarification on the genetic materials used to assess the effect of inbreeding on suppression of the triploid block (see 1. Genetic Materials), the second relates to the connections between DNA methylation changes at genes implicated in the triploid block (see 2. DMRs) and the third pertains to the apparent disconnect between the role of RdDM in gene silencing and the downregulation of genes associated with hypo DMRs in nrpd1 mutants (see 3. Model).

1. 	Genetic Materials: Figure 1 nicely demonstrates that upon inbreeding the osd1nprd1, osd1nrpe1, and osd1drm2 lines are all able to suppress the triploid block.  However, in Fig S2, which outlines the approach for generating these inbred lines, it is not clear what the genotypes of the starting mutants were.  For example, were the nrpd1, nrpe1, rdr2, and drm2 mutant keep heterozygous prior to the initial cross to the osd1 mutant?  If not, when were they taken to homozygosity? This is an important point as recent studies have show that there are some sites in nprd1-/- mutants where methylation cannot be restored by adding back a functional NRPD1 gene (PMID: 32514141), demonstrating that the past history of these lines could be critical. As is done for the bottom portions of the graphics, please indicated the genotypes of the mutants selected for the initial selfing and the first cross to the osd1 mutant.

**Response: We included the information on the genotypes used for the crosses into Figure S2. All mutants were received as heterozygotes from the stock center and propagated for one or two more generations before crossing to osd1. With the exception of rdr2 that was crossed as a heterozygous mutant, the other mutants were crossed after being homozygous. Nevertheless, we do not think that this explains the different behavior of rdr2 compared to nrpd1, since rdr2 also had a weak suppressive effect when used as a tetraploid mutant (Figure S1). As the reviewer correctly points out, previous work revealed that some loci targeted by RdDM fail to become remethylated after loss of RdDM function. However, we do not think that these loci are responsible for the triploid block; since based on our work rather the gradual loss of DNA methylation at some specific loci is linked to the gradual suppression of the triploid block by rddm mutants. Thus, only the loci that can be re-methylated after the cross to WT are likely to be involved. We included this reasoning into the discussion as follows: “Previous work revealed that some loci demethylated in a nrpd1 background do not regain their original methylation level after restoration of NRPD1 function [Li et al., 2020]. The gradual suppression of the triploid block through RdDM mutants, however, strongly suggests that the loci involved in the triploid block can be efficiently reset upon restored RdDM function.”**

2. 	DMRs: To better evaluated the potential significance of the DMRs from leaves extrapolated onto data from the endosperm, additional information about the data processing and analyses are required. Ideally, one would directly test the effects of methylation at these loci over several generations directly in endosperm, but given the difficulties and costs of such assays, it may be outside the scope of the current work.  Nonetheless, additional information about the current data could already be helpful assessing the reproducibility and scale of methylation differences observed at these loci in leaf tissue upon inbreeding.

*  	Replicate Data: Based on the coverage (Table S1) the WT, F2 nrpd1, and Fi nrpd1 were all sufficiently high to treat independently. Are the DMRs presented in Table S2 those that were identified between both WT replicates and either both F2 nrpd1 or both Fi nrpd1 replicates indicating the most reproducible DMRs or are they all the pair-wise DMRs?  For bulk analyses, like Fig. 2G or Fig. S4, including all the pairwise vs the more robust DMRs would likely not make much difference, but for the regions highlighted in Fig. 6B and Fig. S6 it would very informative to know how reproducible these DMRs are. Along these same lines, given the scale of the screen shots shown, its difficult to evaluate the changes in methylation at these DMRs.  I would suggest showing a zoomed in view and labeling the regions with the % methylation in each genotype and displaying the two biological replicates separately.

**Response: The methodological and analytical reproducibility of the DMRs were assessed by analysing the correlation in methylation levels between replicates within the same condition (Figure S7). Based on the high reproducibility between the replicates, we merged the replicates and based on the merged methylation levels defined DMRs. Since we compared different samples with each other, treating the samples independently would have generated multiple DMRs, which would make downstream analysis and comparisons very complicated. In the revised Figures 6 (now Fig. 5) and S6, we now show the individual replicates separately, which shows that the data are highly reproducible. Also as suggested by the reviewer, we improved the screen shots of the selected loci.**

*  	DMR Thresholds: Fig 2F, it seems that DMRs showing very little changes in methylation were included (see bottom 1/5th of the heatmaps) which could introduce a large about of noise in the regions being studied.  The inclusion of DMRs that show minimal differences could also affect the overall conclusions of the study, if for example, the DMRs highlighted in Fig. 6B and Fig. S6 all fall into this category.  Its not clear from the methods how this threshold was set and if it also has a % methylation change cutoff or just a p-value cutoff.  Please clarify the methods and indicate where the DMRs in Fig. 6B and Fig. S6 fall on these heatmaps.

**Response: To avoid including DMRs with minimal differences, we selected only 10% of bins with the highest difference in methylation that passed Fisher’s exact test. We indicated in the methods the selection of DMRs as follows: “Differentially methylated regions (DMRs) in CHG and CHH contexts were defined using 50-bp windows across the genome as units. Only hypomethylation in order wild type > F2 nrpd1 (DMR1), F2 nrpd1 > Fi nrpd1 (DMRi), and wild type > Fi nrpd1 (DMRx) was considered. Windows with differences in fractional methylation below the 1st decile (Fisher’s exact test p-value < 0.01) were selected and these were merged if they occurred within 300 bp (see Additional file 8: Table S7).”**

3. 	Model:

*  	While the RdDM pathway is quite complicated, as are the various other pathways and proteins influencing the triploid block, the fact that genes associated with losses of methylation in nrpd1 mutants are downregulated in the endosperm seems at odds with current models of gene silencing.  Do the authors have a model that would explain this disconnect that could be presented in the discussion?

**Response: Previous work (Martinez et al., 2018, Satyaki et al., 2019) revealed that loss of paternal NRPD1 causes increase of CHH methylation at loci that lose DNA methylation in the endosperm of triploid seeds. We found that many genes losing non-CG methylation in 3x seeds restore non-CG methylation in 3x nrpd1 seeds (Figure 5C). The mechanism causing increase of non-CG methylation upon loss of paternal NRPD1 function and its relevance remains to be explored. We included the following paragraph to the discussion addressing this point: “Successive inbreeding of Arabidopsis ddm1 (decrease in dna methylation1) was shown to trigger enhanced phenotypic defects over generations caused by progressive loss of CG methylation as well as activation of alternative silencing mechanisms (Ito et al., 2015). Similarly, met1 mutants were shown to progressively form new and aberrant epigenetic patterns over generations (Mathieu et al., 2007). In both mutants a retargeting of H3K9me2 was observed, which depends on 21/22-nt easiRNAs (Ito et al., 2015; Lee et al., 2020). Whether the increase of CHH and CHG methylation upon loss of paternal NRPD1 function in the endosperm of triploid seeds is caused by a retargeting of H3K9me2 remains to be tested. It would provide an explanation for the finding that mutants homozygous for components of the RdDM pathway are able to suppress the triploid block (Satyaki et al., 2019).”**

*  	Also the fact that rdr2 mutants fail to suppress the triploid block is at odds most RdDM models (as mentioned in the discussion) and suggest perhaps Pol-IV and another RDR are required to make siRNAs that rather than targeting methylation participated in post-transcriptional gene silencing.  In this case, loss of the downstream RdDM machinery would suppress the triploid block not because of alterations in methylation that affect gene regulation at the transcriptional level, but rather alterations in methylation that are required for Pol-IV targeting. This is alluded to in the discussion, but is not directly presented as an alternative hypothesis to their model wherein losses of methylation lead to the downregulation of specific target genes. Given the lack of transgenerational methylation data in nrpd1 and nrpe1/drm2 endosperm, I think both models should be presented.

**Response: We discussed this alternative hypothesis in the discussion as follows: “We consider two non-mutually exclusive hypotheses that may explain the effect of the RdDM on the triploid block. Through its canonical function, RdDM could affect DNA methylation at key loci, inducing the triploid block. Alternatively, NRPD1-dependent 21/22-nt siRNAs could mediate post-transcriptional gene silencing of key loci, consistent with the recently shown requirement of NRPD1 to produce 21/22-nt siRNAs [22,23].”**

Minor comments

*  	I would suggest adding Table S7 to Table S2.  Also, the numbers of DMRs in the Venn diagrams (Fig. 2) don't match perfectly the numbers in Table S2/S7.  Please check and correct as needed.

**Response: Since both tables contain different type of data (Table S2 lists the merged DMRs and their position; while Table S7 (previous Additional file 7: Table S6, now Additional file 8: Table S7) shows number of DMRs in single 50 bp bins and number of merged DMRs (merged when closer than 300 bp)) we prefer to keep both tables separated. The numbers of DMRs shown in the revised Table S2 correspond to the numbers shown in Figure 2. However, because the Venn diagrams show overlaps of DMRs that differ in length, the numbers of total DMRs shown in Table S7 slightly differ from the numbers shown in Figure 2 and Table S2 (the overlap of a long DMR with two short DMRs will be counted as one DMR in the intersection).**

*  	I found the data presentation in Fig S4A,B was more intuitive than in Fig 4A,B.  As they are showing the same data, just plotted different ways, and take similar space, I would suggest moving the boxplots from Fig. S4 to the main figure.

**Response: Agreed, we moved the boxplots from Fig. S4 to the main figure (now Fig. 3) as suggested.**

*  	Please add information to the methods about how the biological replicates for the ChIPseq data were taken into account for generating the boxplots in Fig. 4C, D.

**Response: We used the mean of three biological replicates for the ChIPseq data shown in Fig. 4C, D (now Fig. 3E, F). The use of triplicates was stated in the methods and the quality of the replicates shown in Figure S8.**

*  	Please cite in the main text sources of genomic data re-analyzed for this study.  For example, in the section covering the transcriptomic data in the endosperm and seeds it is not clear this was previously published data.

**Response: We included the relevant data sources as requested.**

*  	In the main text, ln 230, endosperm of 3x vs 2x seeds are mentioned but in Fig. 6B and Fig. S6 the endosperm tracks are labeled as 3x and 4x.  Please clarify.

**Response: The endosperm of 2x seeds is 3x and the endosperm of a 3x seed when derived from a 2x x 4x cross is 4x. Since we show endosperm-specific data in Figures 6 (now Fig. 5) and S6, we prefer to show the ploidy grade of the endosperm.**

*  	Please add a description of the color bar for Fig. 2F.  I assume it is showing % methylation for each DMR?

**Response: We show fractional methylation levels and added this information to the corresponding figure legends.**

*  	Ln 59  "stablished" should be "established"

**Response: Corrected, thanks.**

*  	Ln 71-72  What is meant by "paternally-biased"?

**Response: Arabidopsis NRPD1 is not defined as imprinted, but the paternal allele is higher expressed than expected.**

*  	Ln 76  Should "eukaryotic TE" be "euchromatic TE"?

**Response: Corrected, thanks.**

Reviewer #2: The manuscript by Wang et al. addresses the molecular mechanisms responsible for triploid block in plants. This phenomenon attracted a lot of recent attention and has been shown to be controlled by RNA silencing. Two groups reported conflicting results about the involvement of particular silencing factors. These inconsistent results greatly affected the ability to interpret existing data. This manuscript resolves this inconsistency and shows that it is caused by a factor previously assumed to be irrelevant - inbreeding of RdDM mutants. This is an important and timely discovery. The manuscript further investigates the impact of inbreeding on accumulating changes in DNA methylation and expression of relevant genes. This aspect of the manuscript combines high quality results with insights that are less complete.

Under more typical circumstances, the preliminary nature of some of the presented work would justify major criticism. In this case, it should however be balanced by the timeliness of this study. The conflicting data have been published less than two years ago and resolving the existing conundrum required performing lengthy inbreeding crosses. The field will greatly benefit from this study being published as soon as possible.

Specific comments

1. DNA methylation has been assayed mostly in leaves while triploid block occurs in developing seeds. The limitations of this approach should at least be openly addressed in the manuscript.

**Response: We added the following statement to the results to justify the use of leaves: “Since CHH methylation levels are developmentally regulated in the endosperm and thus prone to potential variability, we analyzed leaf tissue of F2 and inbred (Fi) nrpd1 mutants.”**

2. The vaguely explained Fi population (more than three times inbred) is not optimal but still acceptable given the time needed to perform inbreeding. It should however be explained in as much detail as possible.

**Response: The nrpd1-3 (NASC N628428) were the T2 or T3 generation according to the information obtained from NASC. We updated Fig S3 to clearly show the genotypes used for the crosses starting from the generation we obtained from the stock center.**

3. Classification of DMRs shown in Fig. 3 offers very little insights into the studied process and could be moved to the supplement (or removed from the manuscript).

**Response: Agreed, moved to the supplement (now Fig. S4).**

4. The observation that loci that progressively lose DNA methylation depend on CMT2 and CMT3 is very interesting but not much is offered in terms of explaining why CMT2 and CMT3 cannot maintain silencing in the absence of RdDM. Addressing this question directly may be difficult without lengthy inbreeding experiments with DNA methyltransferase mutants, however any additional insights based on existing datasets would strengthen the manuscript. A thorough discussion of this issue is needed.

**Response: Our findings are consistent with previous work, showing that RdDM is transient, and loci initially targeted by RdDM transition to more stable silencing maintained by MET1/CMT2/CMT3 (Wang and Baulcome, 2020: doi: 10.1038/s41467-020-14995-6). During the transition step, both complexes might be required to properly methylate those loci and the presence of only one pathway is not sufficient to maintain methylation. We added the following paragraph to the discussion to explain this idea as follows: “In line with previous findings (Wang and Baulcome, 2020), we propose that DMR1 are loci recently targeted by RdDM and that therefore strongly rely on RdDM to maintain DNA methylation, whereas DMRi are possibly ancient RdDM targeted loci transiting from RdDM to an RdDM-independent maintenance phase involving CMT2/CMT3.  This transition state could explain why the CMT2/CMT3 pathway is not sufficient to maintain DNA methylation at those loci.”**

5. Data shown in Fig. 5A do not convince that CHH methylation has no impact on expression of relevant genes. A more in depth analysis not based on multiple arbitrary thresholds would be needed to support this conclusion.

**Response: We addressed this concern by phrasing as follows: “While the overlap of deregulated genes with CHH DMRi was not significant (Fig. 4A), the overlapping genes also included AGLs and several ARFs, similar to deregulated genes overlapping with CHG DMRi (Additional file 4: Table S3).**

6. One label on Fig. 5A is not clear "Genes upregulated in endosperm of 3x and downregulated in 3x nrpd1 seeds". Did the authors mean "… or downregulated…"?

**Response: We rephrased to: ”Genes upregulated in endosperm of 3x vs 2x and downregulated in 3x nrpd1 vs 3x seeds”. (now Fig. 4A)**

7. GO analysis shown in Fig. 5B is not particularly conclusive and should be interpreted carefully.

**Response: We modified the text and emphasized that GO terms including AGLs and ARFs are strongly enriched.**

8. The effects of accumulating changes in DNA methylation on genes that encode proteins, which directly affect endosperm development, although addressed in the manuscript, are not clear. The ability to attribute the observed effects to particular target genes would certainly strengthen this study but may require extensive additional experimental effort. A more direct explanation of the limitations of the obtained insights would help the readers better understand this aspect of the manuscript.

**Response: As we do not have direct evidence on potential targets directly regulated by RdDM in triploid seeds, we shortened this part of the discussion.**

9. The last section of the results is very confusing. It appears to address an important issue of DNA methylation in leaves vs. in seeds. However - as explained above - this issue is not properly explained, and this section comes an unpleasant surprise. It should be reworded and better integrated with the rest of the manuscript.

**Response: We reworded this paragraph to make it easier to follow. We also added explanatory sentences at the end to frame this paragraph and also show the limitations of the current findings: “Thus, genes marked by DMRi in leaves lose DNA methylation in the endosperm of triploid seeds, correlating with increased expression. Conversely, loss of paternal NRPD1 causes increased DNA methylation in the endosperm, correlating with repression of many genes. The mechanism leading to increased DNA methylation upon loss of paternal NRPD1 and whether the effect on gene expression is a direct or rather an indirect consequence remains to be established.”**

10. The results of this study are likely to be relevant beyond the process of triploid block and the manuscript would greatly benefit from putting the obtained results in context of earlier studies where accumulating defects were observed in met1 and ddm1 mutants but not in RdDM mutants.

**Response: To address this concern, we added the following paragraph to the discussion: ”Successive inbreeding of Arabidopsis ddm1 (decrease in dna methylation1) was shown to trigger enhanced phenotypic defects over generations caused by progressive loss of CG methylation as well as activation of alternative silencing mechanisms (Ito et al., 2015). Similarly, met1 mutants were shown to progressively form new and aberrant epigenetic patterns over generations (Mathieu et al., 2007). In both mutants a retargeting of H3K9me2 was observed, which depends on 21/22-nt easiRNAs (Ito et al., 2015; Lee et al., 2020). Whether the increase of CHH and CHG methylation upon loss of paternal NRPD1 function in the endosperm of triploid seeds is contributed by a retargeting of H3K9me2 remains to be tested. It would provide an explanation for the finding that mutants homozygous mutant for components of the RdDM pathway are able to suppress the triploid block (Satyaki et al., 2019).”**

11. It is not clear how duplicated (WGBS) or triplicated (ChIP-seq) datasets were analyzed to demonstrate reproducibility.

**Response: The methodological and analytical reproducibility of the DMRs were assessed by analysing the correlation of methylation levels between replicates within the same condition (Figure S7). Based on the high reproducibility between the replicates, we merged the replicates and based on the merged methylation levels defined DMRs. An analysis of the quality of the ChIP samples is provided in S8. Based on the correlation analysis, the quality of the replicates is very high, justifying an analysis of pooled replicates.**

Reviewer #3:

This submission describes a genetic and molecular analysis of triploid block suppression across multiple generations of inbreeding of several Arabidopsis RdDM mutants. DNA methylation is also analyzed in multiple genotypes across generations. The methods are appropriate and well described, including suitable controls. The work is important in understanding some previously reported discrepancies with regard to the genetic requirement of some factors in suppressing the triploid block. The authors reasonably assert that the differences previously observed are related to the level of inbreeding in the RdDM backgrounds, which differed between the two studies.  This assertion is tested adequately with this work, and the results are fairly interpreted.  This work is likely to be of broad interest to many in the field of gene silencing and provides insight into the role of epigenetic regulation in detection and respond to ploidy during early development.

I have three comments that are worthy of consideration but do not substantially change my opinion of the work.

First, although the overall crossing strategy is clearly described in the supplement figure, it is not clear if the starting stocks were heterozygous or homozygous insertion lines, which might mean that the individuals crossed with osd1 had experienced homozygosity of the RdDM mutations for differing numbers of generations.  This may or may not change anything about the interpretation of the reported results, but give the apparent importance of inbreeding, it would be useful to describe this clearly.  Also, although it may be obvious for scientists very close to this work, it would be nice to state if all of the mutants utilized contain null mutations for the genes of interest.

**Response: We included the information on the genotypes used for the crosses into Figure S2. All mutants were received as heterozygotes from the stock center and propagated for one or two more generations before crossing to osd1. With the exception of rdr2 that was crossed as a heterozygous mutant, the other mutants were crossed after being homozygous. Nevertheless, we do not think that this explains the different behavior of rdr2 compared to nrpd1, since rdr2 also had a weak suppressive effect when used as a tetraploid mutant (Figure S1). As the reviewer correctly points out, previous work revealed that some loci targeted by RdDM fail to become remethylated after loss of RdDM function. However, we do not think that these loci are responsible for the triploid block; since based on our work rather the gradual loss of DNA methylation at some specific loci is linked to the gradual suppression of the triploid block by rddm mutants. Thus, only the loci that can be re-methylated after the cross to WT are likely to be involved. We included this reasoning into the discussion as follows: “Previous work revealed that some loci demethylated in a nrpd1 background do not regain their original methylation level after restoration of NRPD1 function [Li et al., 2020]. The gradual suppression of the triploid block through RdDM mutants, however, strongly suggests that the loci involved in the triploid block can be efficiently reset upon restored RdDM function..”**

**We also added a statement into the methods that the used alleles are null alleles.**

Second, with regard to the data reported in Figure 1 for seed germination and abortion, it is not completely clear if the two panels of data represent mutually exclusive seed populations.  In other words, is it % germination of all seed (including normal and aborted) or of normal seed only?  Also, n (number of seed for each analysis) is not indicated for any of this data (aside from a note about the number of siliques constituting a biological replicate).

**Response: In Figure1, the frequencies of abortion and germination were calculated including normal and aborted seeds. Number of seeds for each analysis was added to Figure 1, and the legends were improved accordingly.**

Thirdly, related to the second comment, the figure legends are not very detailed and are missing some potentially useful information.  Including information about what data is actually plotted, all acronyms used should be explained in figure legends but in many cases are not.  For example, in the legend for figure 2, it is stated that DMRs is an acronym for differentially methylated regions; the use of an 's' in the acronym here is a little confusing given the very specifically defined group of differentially methylated regions designated as DMR1s in the results section.  DMR1s and other designations are also used in Figure 3, without any explanation in the legends.  I also do not see much information about how the TEs analysis was completed, or at least a citation for the definition of the different families.

**Response: Thanks for pointing this out. To avoid confusion, we modified DMR1s to ‘DMR1spec’. The lacking information was updated accordingly in the manuscript.**

**Second round of review**

**Reviewer 1**

In the revised manuscript Wang et al. added the requested information to clarify their experimental design and data analysis pipelines.   They also modified the text to include alternative models involving post-transcriptional regulation in connection to the triploid block.   These modifications, along with those requested by other reviewers, improved the manuscript and I have no additional comments.

**Reviewer 2**

I am satisfied with the revisions and authors' responses to my comments.
